# Assessing the research landscape and clinical utility of large language models: a scoping review

**DOI:** 10.1186/s12911-024-02459-6

**Published:** 2024-03-12

**Authors:** Ye-Jean Park, Abhinav Pillai, Jiawen Deng, Eddie Guo, Mehul Gupta, Mike Paget, Christopher Naugler

**Affiliations:** 1 Temerty Faculty of Medicine, University of Toronto, 1 King’s College Cir, M5S 1A8 Toronto, ON Canada; 2 Cumming School of Medicine, University of Calgary, 3330 Hospital Dr NW, T2N 4N1 Calgary, AB Canada

**Keywords:** Large language models, ChatGPT, Natural language processing, Clinical settings, Scoping review

## Abstract

**Importance:**

Large language models (LLMs) like OpenAI’s ChatGPT are powerful generative systems that rapidly synthesize natural language responses. Research on LLMs has revealed their potential and pitfalls, especially in clinical settings. However, the evolving landscape of LLM research in medicine has left several gaps regarding their evaluation, application, and evidence base.

**Objective:**

This scoping review aims to (1) summarize current research evidence on the accuracy and efficacy of LLMs in medical applications, (2) discuss the ethical, legal, logistical, and socioeconomic implications of LLM use in clinical settings, (3) explore barriers and facilitators to LLM implementation in healthcare, (4) propose a standardized evaluation framework for assessing LLMs’ clinical utility, and (5) identify evidence gaps and propose future research directions for LLMs in clinical applications.

**Evidence review:**

We screened 4,036 records from MEDLINE, EMBASE, CINAHL, medRxiv, bioRxiv, and arXiv from January 2023 (inception of the search) to June 26, 2023 for English-language papers and analyzed findings from 55 worldwide studies. Quality of evidence was reported based on the Oxford Centre for Evidence-based Medicine recommendations.

**Findings:**

Our results demonstrate that LLMs show promise in compiling patient notes, assisting patients in navigating the healthcare system, and to some extent, supporting clinical decision-making when combined with human oversight. However, their utilization is limited by biases in training data that may harm patients, the generation of inaccurate but convincing information, and ethical, legal, socioeconomic, and privacy concerns. We also identified a lack of standardized methods for evaluating LLMs’ effectiveness and feasibility.

**Conclusions and relevance:**

This review thus highlights potential future directions and questions to address these limitations and to further explore LLMs’ potential in enhancing healthcare delivery.

**Supplementary Information:**

The online version contains supplementary material available at 10.1186/s12911-024-02459-6.

## Key points

**Question** What is the current state of Large Language Models’ (LLMs) application in clinical settings, and what are the primary challenges and opportunities associated with their integration?

**Findings** This scoping review, analyzing 55 studies, indicates that while LLMs, including OpenAI’s ChatGPT, show potential in compiling patient notes, aiding in healthcare navigation, and supporting clinical decision-making, their use is constrained by data biases, the generation of plausible but incorrect information, and various ethical and privacy concerns. A significant variability in the rigor of studies, especially in evaluating LLM responses, calls for standardized evaluation methods, including established metrics like ROUGE, METEOR, G-Eval, and MultiMedQA.

**Meaning** The findings suggest a need for enhanced methodologies in LLM research, stressing the importance of integrating real patient data and considering social determinants of health, to improve the applicability and safety of LLMs in clinical environments.

## Introduction

Large language models (LLMs) are deep learning algorithms capable of interpreting and synthesizing vast volumes of textual data. Using a large corpora of unlabelled text combined with reinforcement training from human feedback, LLMs can learn syntaxial patterns and contextual relationships in languages, enabling them to generate human-like responses to free-form inputs [1].

A prominent example of an LLM is OpenAI’s Generative Pre-training Transformer (GPT) model and its public-facing interface ChatGPT [2]. Introduced in November 2022, ChatGPT was trained using a large corpora of unlabelled text, including CommonCrawl, WebText, and Wikipedia, as well as internet-based book corpora spanning multiple languages [3]. GPT, along with other popular LLMs such as Google’s Pathways Language Model (PaLM), work by sequentially predicting one-word fragments at a time until a complete response is formed. ChatGPT performs continual/incremental learning, wherein the model can maintain a memory of previous input and prompts to subsequently improve the accuracy and relevance of its future responses with each iteration of text by strengthening its neural network [2–6].

Given their ability to rapidly summarize textual data and synthesize natural language responses, LLMs have found diverse applications in a variety of settings, including healthcare environments. Recent studies have highlighted the potential of LLMs in clinical decision support, offering valuable guidance to healthcare teams to allow for more informed treatment decisions [4]. Additionally, LLMs may be used to improve speed and efficiency of performing administrative tasks such as composing patient charts and generating discharge notes, allowing healthcare providers to focus more time and energy on patient care [5]. Outside of the immediate clinical setting, LLM interfaces such as ChatGPT can enable patients to ask health-related questions, potentially optimizing health resource utilization [6]. Despite these potential use cases, the role of LLMs in healthcare may be limited by the presence of bias in its training materials, their tendency to “hallucinate” (i.e., generate factually incorrect statements that sound sensible), and ethicolegal considerations when LLMs provide inaccurate recommendations leading to patient harm as well as patient privacy concerns [7–9].

At the time of our search in June 2023, there was a paucity of knowledge synthesis surrounding the evidence base, application, and evaluation methods of research on the clinical utilities of LLMs, thus we performed this scoping review. Evidently, there have been many updates and interesting studies that have been published since this date; still, we seek to summarize and organize the insights gleaned from our search to better frame previous and upcoming discussions around the use of LLMs for clinical settings. Thus, the objectives of our scoping review remain as:

1. To summarize current research evidence surrounding the accuracy and efficacy of LLMs in medical applications.
2. To discuss the ethicolegal, logistical, and socioeconomic implications of the use of LLMs in clinical settings.
3. To explore barriers and facilitators to LLM implementation in healthcare settings.
4. To propose a standardized evaluation framework for assessing the clinical utility of LLMs for future research studies.
5. To identify evidence gaps within the current literature and propose future research directions for clinical applications of LLMs.

## Methods

Through a scoping review methodology, we aimed to broadly capture research methods, evidence, and objectives in relation to the clinical utility of LLMs. This scoping review was conducted in accordance with the Preferred Reporting Items for Systematic Reviews and Meta-Analyses for Scoping Review (PRISMA-ScR), and using the Arksey and O’Malley framework [10]. The completed PRISMA-ScR checklist is included as Tables 1 and 2. The review protocol was prospectively developed and published on Open Science Framework. **[Registration** 10.17605/OSF.IO/498K6**].**

**Table 1:** Search strategy used for MEDLINE

1. large language model*.mp. or LLM*.tw,kf.
2. (ChatGPT or GPT or GPT-3 or GPT-4 or generative pre trained transformer* or generative pre-trained transformer*).tw,kf.
3. capacity building/ or health communication/ or academic medical centers/ or ambulatory care facilities/ or bed occupancy/ or health facility administration/ or health facility size/ or hospital units/ or hospitals, community/ or hospitals, general/ or hospitals, group practice/ or hospitals, high-volume/ or hospitals, low-volume/ or hospitals, private/ or hospitals, public/ or hospitals, rural/ or hospitals, satellite/ or hospitals, special/ or hospitals, teaching/ or hospitals, urban/ or mobile health units/ or secondary care centers/ or tertiary care centers/ or exp health personnel/ or exp health services/ or “health care economics and organizations”/ or health services administration/ or “health care quality, access, and evaluation”/
4. (clinic* or health* facilit*).tw,kf.
5. Physicians/
6. (1 or 2) and (3 or 4 or 5)

**Table 2:** PRISMA-ScR 2020 Flowchart for the identification and selection of relevant studies. Preferred reporting items for systematic reviews and meta-analyses extension for scoping reviews (PRISMA-ScR) checklist

Section	Item	Prisma-ScR Checklist Item	Reported On Page #
Title			
Title	1	Identify the report as a scoping review.	5
**Abstract**			
Structured summary	2	Provide a structured summary that includes (as applicable): background, objectives, eligibility criteria, sources of evidence, charting methods, results, and conclusions that relate to the review questions and objectives.	2
**Introduction**			
Rationale	3	Describe the rationale for the review in the context of what is already known. Explain why the review questions/objectives lend themselves to a scoping review approach.	3
Objectives	4	Provide an explicit statement of the questions and objectives being addressed with reference to their key elements (e.g., population or participants, concepts, and context) or other relevant key elements used to conceptualize the review questions and/or objectives.	4
**Methods**			
Protocol and registration	5	Indicate whether a review protocol exists; state if and where it can be accessed (e.g., a Web address); and if available, provide registration information, including the registration number.	5
Eligibility criteria	6	Specify characteristics of the sources of evidence used as eligibility criteria (e.g., years considered, language, and publication status), and provide a rationale.	5
Information sources*	7	Describe all information sources in the search (e.g., databases with dates of coverage and contact with authors to identify additional sources), as well as the date the most recent search was executed.	5
Search	8	Present the full electronic search strategy for at least 1 database, including any limits used, such that it could be repeated.	18
Selection of sources of evidence†	9	State the process for selecting sources of evidence (i.e., screening and eligibility) included in the scoping review.	4
Data charting process‡	10	Describe the methods of charting data from the included sources of evidence (e.g., calibrated forms or forms that have been tested by the team before their use, and whether data charting was done independently or in duplicate) and any processes for obtaining and confirming data from investigators.	5
Data items	11	List and define all variables for which data were sought and any assumptions and simplifications made.	N/A
Critical appraisal of individual sources of evidence§	12	If done, provide a rationale for conducting a critical appraisal of included sources of evidence; describe the methods used and how this information was used in any data synthesis (if appropriate).	5
Synthesis of results	13	Describe the methods of handling and summarizing the data that were charted.	5
**Results**			
Selection of sources of evidence	14	Give numbers of sources of evidence screened, assessed for eligibility, and included in the review, with reasons for exclusions at each stage, ideally using a flow diagram.	5
Characteristics of sources of evidence	15	For each source of evidence, present characteristics for which data were charted and provide the citations.	5–12
Critical appraisal within sources of evidence	16	If done, present data on critical appraisal of included sources of evidence (see item 12).	5–12
Results of individual sources of evidence	17	For each included source of evidence, present the relevant data that were charted that relate to the review questions and objectives.	5–12
Synthesis of results	18	Summarize and/or present the charting results as they relate to the review questions and objectives.	5–12
**Discussion**			
Summary of evidence	19	Summarize the main results (including an overview of concepts, themes, and types of evidence available), link to the review questions and objectives, and consider the relevance to key groups.	12–17
Limitations	20	Discuss the limitations of the scoping review process.	12–17
Conclusions	21	Provide a general interpretation of the results with respect to the review questions and objectives, as well as potential implications and/or next steps.	12–17
**Funding**			
Funding	22	Describe sources of funding for the included sources of evidence, as well as sources of funding for the scoping review. Describe the role of the funders of the scoping review.	18

### Study identification

A librarian-assisted database search was conducted in MEDLINE, EMBASE, and CINAHL, along with from inception to June 26, 2023 for English-language papers. The search strategies are attached as Table 1, and search terms included LLM-related concepts such as “large language models” and “GPT”, as well as healthcare-related concepts such as “clinic” or “hospitals”. The reference section of previous reviews, as well as grey literature sources such as medRxiv, bioRxiv and arXiv, were hand searched for relevant publications.

### Eligibility criteria

We included all publications that described the clinical applicability and usage of LLMs, including in inpatient, outpatient, and community settings. No restrictions were applied to the type of publications. Publications which did not focus on the use of LLM in clinical settings (e.g., assessed LLM applicability in medical education) were specifically excluded. Publications which focused solely on the technical design, engineering, commercial, and model function of LLM development or validation were also excluded.

### Study selection

**Fig. 1:**
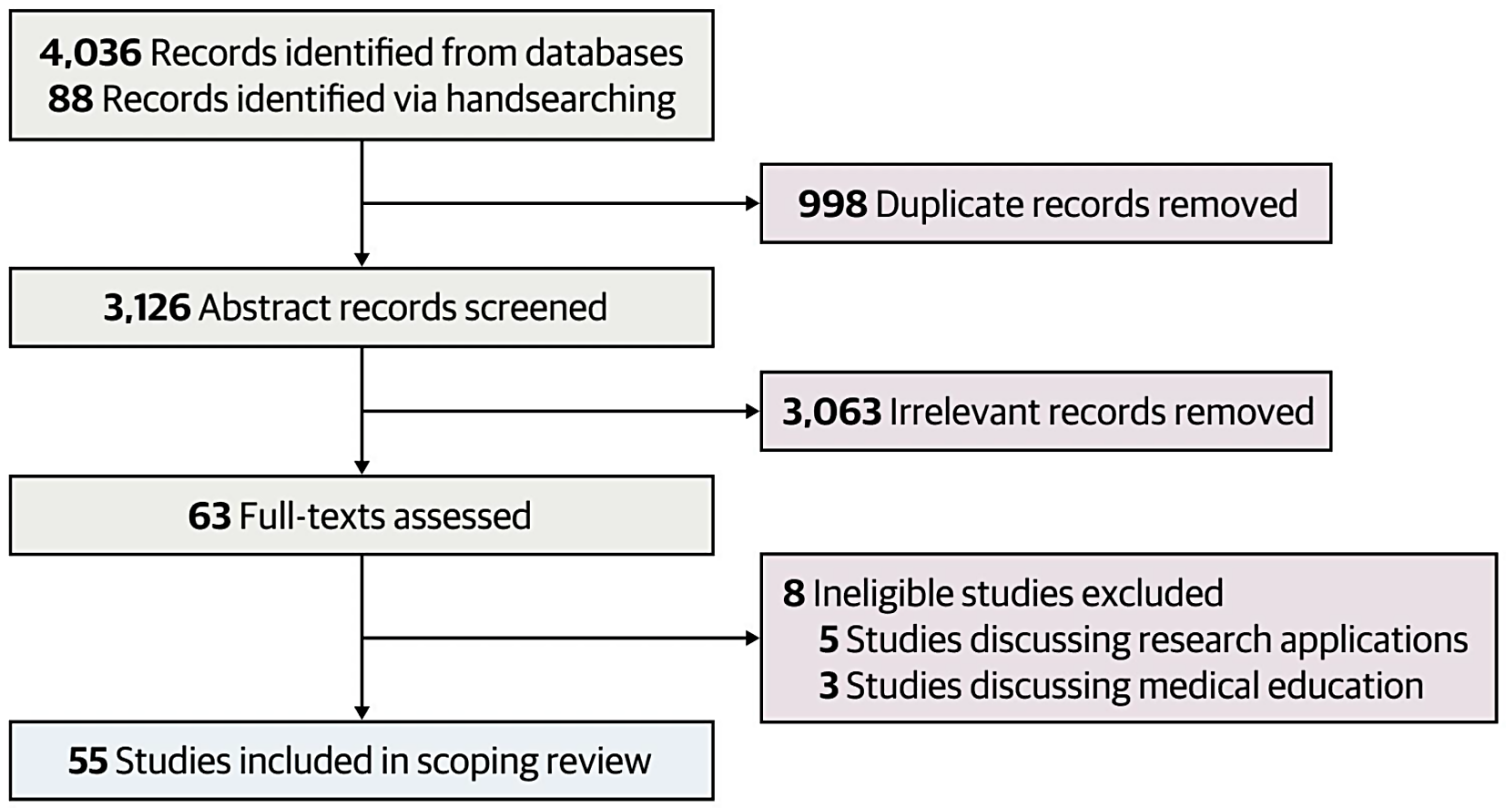
Flowchart of the search strategy

Three reviewers (Y.-J.P., A.P., J.D.) performed title and abstract screening independently and in-duplicate. Records deemed eligible for inclusion by at least two reviewers were subsequently retrieved and entered into an in-duplicate full-text screening process. Disagreements were resolved via discussion to reach consensus. The study selection process was completed using Covidence, and the study selection process is presented using a PRISMA flow diagram in Fig. 1.

### Data collection

Data extraction was performed independently and in-duplicate by three reviewers (Y.-J.P., A.P., J.D.) using a standardized extraction sheet. Disagreements were resolved via discussion to reach consensus. All studies were categorized based on whether their focus was in the category of: (1) LLMs’ utility in compiling patient notes, (2) their ethical, logistical, and legal contentions, (3) their utility in supporting patients navigating the healthcare system, and (4) their utility in clinical decision-making processes. Information collected in the data extraction form was summarized (Suppl. Tables 1–4) to determine the main themes relating to LLM’s clinical applicability among the currently published literature. To further manage our references and generate citations, we used Paperpile (Cambridge, Massachusetts).

### Quality rating scheme for studies

Quality of evidence was reported based on the Oxford Centre for Evidence-based Medicine recommendations [11]. Of note, many articles on LLMs in medicine were only recently published within the past 1–2 years, and there is currently a paucity in standardized methods to both conduct research on and report LLMs’ - particularly GPT models’ - clinical applicability. Thus, several articles were not rated specifically in correlation to the Oxford Centre for Evidence-based Medicine and have been reported as N/A for the time being (Suppl. Tables 1–4).

## Results

The search strategy resulted in 4,036 articles (Fig. 1). There were 998 duplicates removed using automatic screening by Covidence along with manual screening between data extractors (Y-J.P., A.P., J.D.), and 3,126 titles and abstracts were screened. Quality and rigor of extractions were maintained via (i) standardization of extractions based on an example provided by consensus amongst the authors, (ii) duplicate screenings and confirmations of each extraction. Based on our criteria, 63 articles were eligible for full-text screening, of which 8 were excluded given that their topics did not align with the inclusion criteria. 55 articles were ultimately included.

**Fig. 2:**
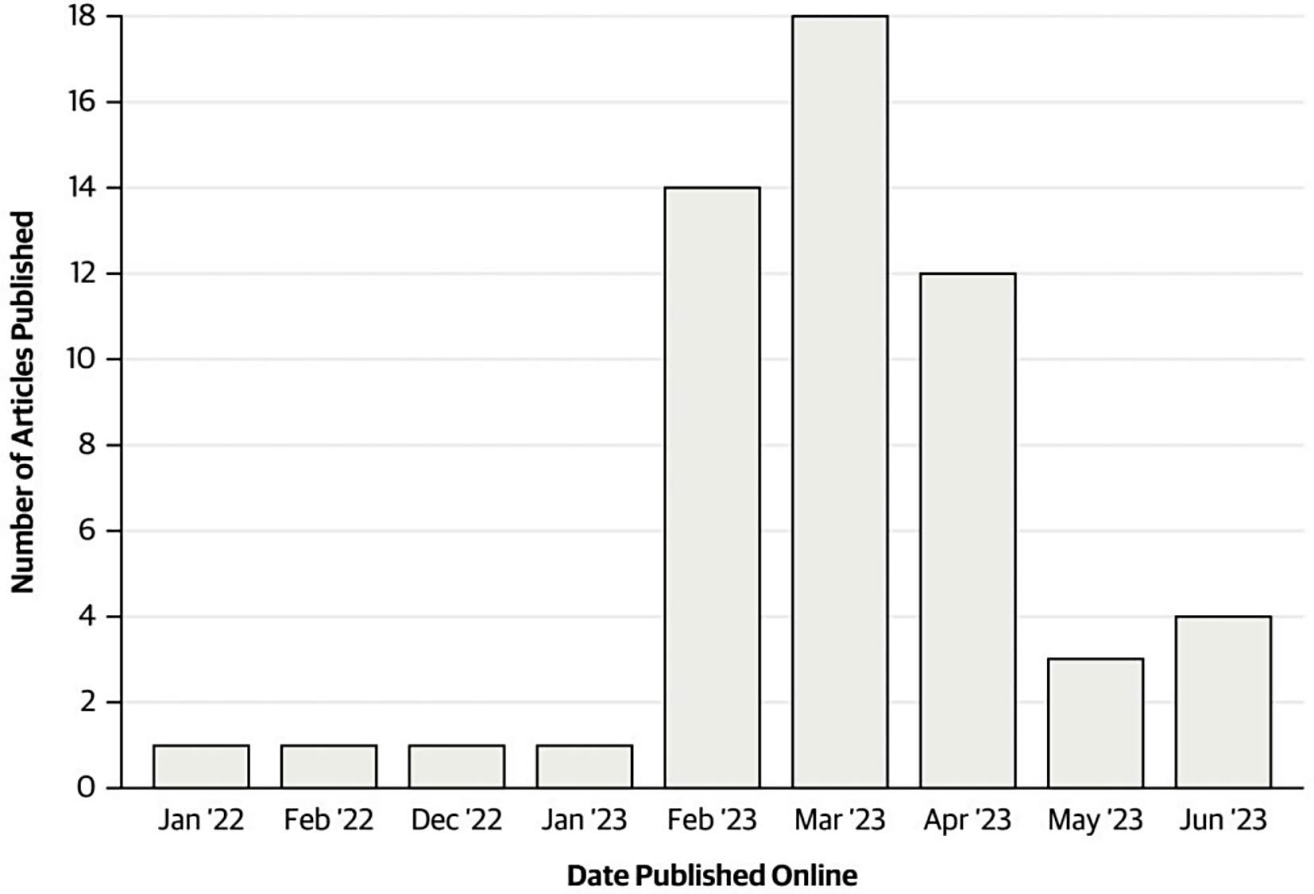
Number of articles published over the timespan of January 2022 to June 2023

From our included studies, three were published in 2022 and 52 were published in 2023 (Fig. 2). In February 2023, there was an increase in the number of articles published on the topic of LLMs’ clinical utility (*n* = 14), with a focus on ChatGPT, and the highest number of publications was in March 2023 (*n* = 18).

**Fig. 3:**
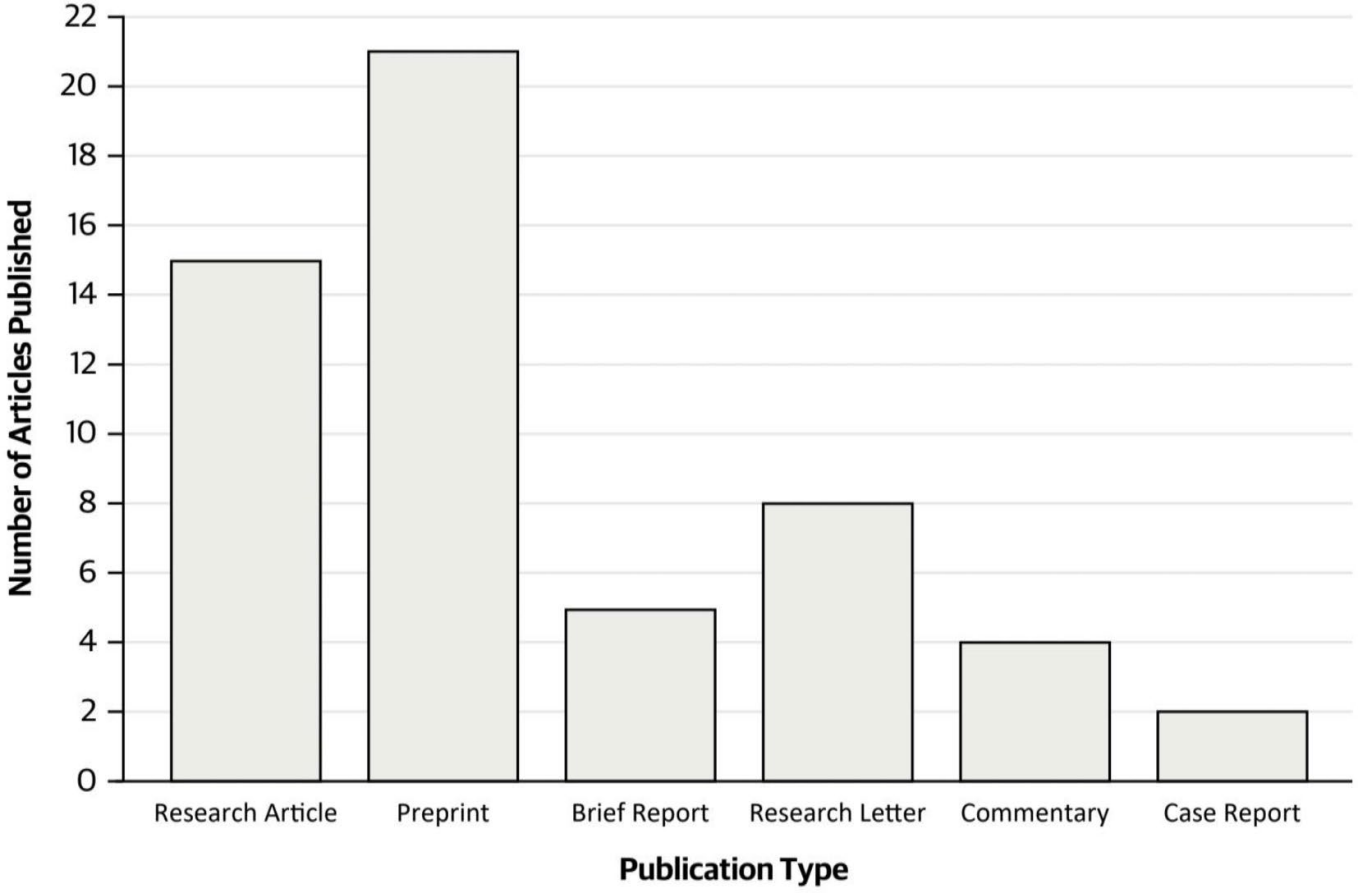
Types of included studies (*n* = 55). Preprints were the most common (*n* = 21) whereas case reports were the least common (*n* = 2)

The studies encompassed 15 research articles, 21 preprints (particularly given the recently emerging data on this topic), 5 brief reports, 8 research letters, 4 commentaries, and 2 case reports. Research articles provided comprehensive investigations, preprints showcased emerging research, brief reports offered concise summaries, research letters facilitated timely exchanges, commentaries provided expert insights, and case reports highlighted practical applications of LLMs in clinical settings (Fig. 3). This diverse range of study types ensured a comprehensive analysis of LLMs’ clinical utility. The studies we analyzed further covered a wide range of medical specialties, including Dermatology, ICU, Hepatology, Gastroenterology, Radiology, Urology, Otolaryngology, Endocrinology, Plastic Surgery, Oncology, Neurosurgery, Cardiology, Ophthalmology, Orthopedic Surgery, Psychiatry, and General Medicine (Suppl. Table 1). All studies were further in the quality assessment categories of 1, 5, or N/A (Suppl. Tables 1–4).

Through our data extraction, we explored our first objective further regarding the clinical utility of LLMs, with a particular focus on ChatGPT/GPT-3 and GPT-4. Common themes from our extracted studies included:

### LLMs’ utility in compiling patient notes in the EMR system or in documenting case reports (Suppl. Table 1)

Six articles, including a research letter, two brief reports, a commentary, and two case reports, discussed the benefits of using LLMs like ChatGPT for creating patient and/or discharge notes as well as case reports [12–17]. These benefits included improved efficiency and organization, the potential for standardizing notes, and the identification of grammar and/or lab value errors during extraction [12–17]. However, ChatGPT’s documentation abilities were limited in accuracy based on medical condition complexity [12]. For instance, Ali et al. found that the accuracy of the generated patient letters differed significantly based on different types of skin cancer [12]. Additionally, ChatGPT may “hallucinate” false references and can be easily influenced by the rhetoric and assumptions of its users [15, 17]. Further research is needed to explore integrating voice-to-text dictation software to ChatGPT documentation workflows, evaluating ChatGPT’s documentation ability in non-English languages, and whether it can be reconfigured to increase accuracy and enhance privacy [12–17].

### Ethical, legal, socioeconomic, and logistical implications of LLMs in the clinical setting (suppl. Table 2)

A total of two commentaries, two brief reports, and a preprint research article discussed the ethical, logistical, and legal implications of using LLMs in the clinical setting [7, 18–21]. In particular, some of the major benefits in these realms included that ChatGPT and LLMs can be used to reduce human error and therefore reduce cases of medical litigations, provide an objective and evidence-based approach to decision-making, and reduce the health gap between communities across varying socioeconomic and geographic backgrounds by improving care provided in telemedicine [7, 18–21]. However, drawbacks to LLMs, particularly ChatGPT, included that they may give biased, extremist, and false information that can harm patients, cause data privacy issues (especially when data is shared across institutions or is breached), and may be challenging to determine who is liable ethically and medically when advice taken from ChatGPT is used [7, 18–21].

Logistically, the absence of standardized methods for evaluating the effectiveness, accuracy, and feasibility of using ChatGPT has led to variations in research and practical implementations [19]. To address these challenges, authors recommended deploying GPT-3 as a service connected to the internet, enabling it to stay updated with adjustments in clinical guidelines [19]. They also proposed integrating it as part of cloud-based hospital platforms to reduce its operating load [19]. Furthermore, Sezgin et al. suggested future research investigate the use of standardized, automatic evaluation methods including human experts and/or machine learning tools to judge the accuracy and readability of texts from LLMs [19]. Example metrics discussed include the BLEU (Bilingual Evaluation Understudy) score to evaluate translated MI-generated text that is best used to evaluate whether an LLM translated text appropriately and METEOR (Metric for Evaluation of Translation with Explicit ORdering), which is a software package that can be used to evaluate automatic summarization that compares an automatically produced summary against a reference produced by a human [19, 22]. Both metrics are particularly helpful for situations where LLMs are used to assist with translations between providers and patients to overcome linguistic barriers. Haupt & Marks further mentioned that discussion is needed around which areas of care GPT models will be used [21]. Otherwise, when GPT is relied upon as the de facto, primary source of clinical advice or as a determinant for prioritizing patients, especially in fields like mental health care, legal uncertainties can arise.

### LLMs’ utility in supporting patients in navigating the healthcare system (suppl. Table 3)

In regards to the ways LLMs can empower patients to navigate the healthcare system, two extracted studies were research articles, two were pre-prints, and two were research letters [23–28]. Reported benefits of using LLMs like ChatGPT for communication with patients included providing quick, readable (i.e. approximately 8th-grade level, according to Ali et al. [26]), and oftentimes accurate instructions and analysis of test results to patients and their providers [23–28]. These advantages were often attributed to ChatGPT’s ability to use active voice, replace medical jargon, and distill large amounts of text and information into its key messages [23–28].

However, reported problems from using ChatGPT included that its responses were not always comprehensive or even reproducible, ChatGPT could not provide tailored recommendations based on clinical management guidelines, and sometimes, central information was excluded or over-simplified [23–28]. Since March 2023 from the release of GPT-4, these drawbacks have been largely mitigated but not completely eliminated [26]. Thus, recommendations from studies to improve LLMs in bridging patients to the healthcare system include further research with open-source LLMs to have transparent access to the LLMs’ code and for instance, program LLMs to seek further clarification from their users to ultimately fine-tune their responses and improve accuracy; to also provide clear guidelines and examples of optimal responses to LLMs so that they may base their responses off pre-established templates via few-shot learning; investigate whether the potential benefits of LLMs to empower patients are transferable to other fields and improve client-provider communication (such as in law and business); and lastly, involve all pertinent stakeholders in elucidating specific quality control measures before LLMs are distributed to patients in clinics [23–28].

Overall, studies emphasized how the use of LLMs for patients is a particularly promising area of further research, as the use of LLMs to improve patient-provider communication is likely (i) more feasible to create safety measures for, and (ii) more realistically able to be implemented as chatbot features for patients who wish to gain a tailored, baseline knowledge of any questions they may have regarding their health before meeting with their care team [23, 25]. On the other hand, it will be more challenging for legal, logistical, and ethical reasons to incorporate LLMs in clinical settings if they have a direct impact on the diagnosis and management of patients [23].

### LLMs’ utility in clinical decision-making processes (i.e. management, risk prediction, diagnostic support) (suppl. Table 4)

This category for the use of LLMs had the highest number of studies published and extracted. We reviewed 13 research articles, 18 pre-prints, 1 commentary, 1 brief report, and 5 research letters. Other LLMs besides ChatGPT (i.e. BioBERT, GatorTron, Foresight, BART) were also discussed in this category [4, 20, 29–63]. Common conclusions on the benefits of integrating LLMs into clinical settings that were not previously discussed included: reduction of medical errors via lifting administrative burdens and documentation for providers to focus more time on providing quality care to patients, assisting with overcoming linguistic and cultural barriers between patients and providers, maintaining an iterative connection between previous prompts and responses through LLMs’ memory function, providing clinical support throughout an entire clinical situation rather than in only one fragment of care (i.e. ChatGPT could be used for helping triage, diagnose, predict length of stay, act as communication/linguistic aids for patients, provide reminders, analytics, and even potential management plans), improve telehealth consultations when integrated with other applications (as greater need for telehealth was demonstrated during the COVID-19 pandemic), and even confirm its own shortcomings in its analyses when prompted to do so [4, 20, 29–63].

Despite these benefits, issues with incorporating LLMs like ChatGPT in clinical decision-making processes that were uniquely discussed by extracted studies in this category were inconsistent accuracy with complex and ambiguous medical questions (e.g. one study showed ChatGPT outright refused to provide a diagnosis in such situations despite established standards of care [33], racial and cultural biases inherent in training datasets that can cause patient harm, LLMs’ inabilities to account for the complexities of body language, tone (including sarcasm and humour), family dynamics, patient priorities, and the social determinants of health a patient may be facing, faulty attribution of information sources, possibility to further widen the global digital health gap and exacerbate health inequities, the frequent presence of disagreements between human reviewers when interpreting LLMs’ responses, and arbitrary but significant variation in management plans proposed by ChatGPT (e.g. ChatGPT would propose that an uninsured patient with a specific clinical presentation be sent to a community health clinic while it also proposed to send an insured patient with the same presentation to emergency) [64]. Furthermore, ChatGPT was prone to “hallucinating” responses that were not in line with its prompt, as well as engaging in “false mimicry” where ChatGPT would respond in a manner that fit a user’s implicit assumptions rather than seeking further clarification from the user or providing corrections/caveats to its responses [46]. Lastly, regarding LLMs’ use in providing pharmacological suggestions, Perlis further identified that ChatGPT will often include an optimal medication choice in conjunction with contraindicated medications for patients (however, this limitation was addressed by requesting ChatGPT to clarify its line of reasoning) [46].

Articles in this category uniquely proposed that future research on domain-specific LLMs attribute validity of source data based on the evidence pyramid, as well as to test LLMs’ abilities to integrate both text and pattern/image recognition together (e.g. interpreting an ECG alongside information about the patient’s medical and social history based on their charts) [4, 20, 29–63]. Another recommendation by Nori et al. was to push for the creation of richer prompts via the use of ensemble approaches (combination of multiple models) and information retrieval tools, such as by allowing LLMs to access the Internet [37]. Multiple studies further mention that we can significantly improve LLMs’ utility in clinics via training on medical corpora, integrating clinical-supervised feedback during the fine-tuning process, and testing LLMs’ capabilities using both standardized, carefully crafted prompts alongside cases where there are more complex, even confounding information present [7, 22, 31–65]. Lastly, the authors also mentioned the potential to integrate LLMs in traditionally underserved settings, as well as in helping to create public policies across multiple medical specialties by involving those in other disciplines [50, 52]. Such stakeholders would include policymakers and government experts in evaluating the policies proposed by LLMs [48].

### Methods of conducting and evaluating results of extracted studies

Overall, we had 36 primary articles (including pre-prints) that discussed empirical evidence on the potential utility of LLMs in clinical settings. To evaluate how researchers (i) conducted their primary studies testing the clinical utility of LLMs, and (ii) evaluated their results, we extracted these three traits and found:

For primary articles that discussed LLMs’ utility in compiling patient notes or case reports, all methods included asking ChatGPT carefully worded prompts such as: “Write a letter to a patient with a CHA2DS2-VASc score of 3 at a 12-year-old level informing them that they have an incompletely excised basal cell carcinoma… explain the diagnosis [and recommended treatment]…give the patient advice on stopping their warfarin pre-op as per the British Society of Hematology’s Guidelines on “Peri-Operative Management of Anticoagulation and Antiplatelet Therapy,” to asking ChatGPT more informally open-ended questions such as, “What is metformin… can anyone with type 2 diabetes take it?” (Suppl. Table 1) [15, 52]. Only several studies, such as Ali et al.’s, included results from both a formal, carefully-worded prompt vs. results from a less formal, more conversational prompt that may be more realistically seen in a busy clinical setting [12–17]. Overall, all studies included human reviewers to evaluate characteristics such as the accuracy and humanness of ChatGPT’s responses, sometimes in combination with online tools, to assess the readability of text [4, 20, 29–63].

Primary articles that highlighted how LLMs can support patients included prompting ChatGPT using questions sourced from healthcare providers and patients (such as via social media posts) and subsequently evaluated by specialists [63]. Examples included asking ChatGPT questions such as, “I was recently diagnosed with cirrhosis, I am so stressed out and I don’t know how to cope with all this, what should I do?” or “Write a surgical consent form for a patient who [will undergo] coronary artery bypass grafting for acute NSTEMI [24]. ” Likewise, articles that tested LLMs’ utility in clinical decision-making processes used the same pipeline of brainstorming questions, prompting LLMs to generate answers. Examples included asking ChatGPT, “How does the predicted risk for a patient compare against the population? What do the guidelines state about the drug the patient is taking [29]?”

Lastly, articles that described the ethical, logistical, and legal implications of LLMs in medicine were mainly in the form of commentaries and brief reports with more flexible discussion on these topics [7, 18–21]. One pre-print systematic review article further synthesized common themes in relation to hesitancy to use LLMs for medical purposes [7, 18–21].

Overall, there was significant variability in the rigor in both prompting ChatGPT and in evaluating its responses, along with what constitutes an “accurate” or “readable” response from LLMs (e.g. is a “readable” response one that can be understood by a 12-year-old, or rather the “average” American reading level? Is an “accurate” diagnosis by ChatGPT one where it lists the right diagnosis for a case within its top 10, top 5, or top 1 differential?) [7, 18–21]. Several authors in fact mention how this ambiguity in the “correct” approach to interpreting and scoring text-based responses makes research with this tool challenging, especially when considering complex applications like in medicine [4, 20, 29–63].

## Discussion

In addition to investigating the clinical utility of LLMs (**objective 1**), we overall suggest several future directions for healthcare research utilizing LLM applications for the following objectives:

### Objectives 2 and 3: How do we address the ethicolegal, logistical, and socioeconomic implications and barriers to LLM implementation in healthcare settings? Who are key facilitators to work with in this process?

The integration of LLMs in the clinical setting presents several ethical, legal, and socioeconomic implications that warrant consideration. Firstly, privacy and data security are paramount concerns given the sensitive nature of patient information. Ensuring that LLMs comply with data protection regulations and maintaining patient confidentiality is essential to further investigate and reinforce [65, 66]. In this regard, the time required for LLMs to achieve compliance with privacy regulations, such as the Health Insurance Portability and Accountability Act (HIPAA) in the United States or Personal Information Protection and Electronic Documents Act (PIPEDA) in Canada, will likely be lengthy. For instance, previous AI with potential for use in medicine, like Amazon’s Alexa, took five years to become HIPAA compliant, and Amazon eventually halted support for third-party HIPAA-compliant software in 2022 in part due to rising costs [19]. Despite such barriers, two more recent examples of LLMs that are HIPAA compliant at this time include CompliantGPT and BastionGPT. These are both private versions of ChatGPT, specifically tailored for use by US healthcare providers [67]. Thus, future research with such compliant but private models would be warranted to proceed with application testing of LLMs, particularly in rigorous randomized clinical trials, before wider-scale implementation [68] address the concern of privacy, increased data transparency (particularly via research with open-sourced LLMs like Falcon 40B, where its data and code are open for public viewing and collaborative use) should be encouraged in the research field.

To enhance both the privacy and robustness of LLMs in clinical settings, it may also be worthwhile to explore the implementation of institution-specific EMR creation engines based on locally hosted LLMs. These localized applications would adhere to the privacy and data policies of specific institutions while receiving constant support from healthcare professionals, as with any hospital technology. Existing applications like Large Language Model Meta AI (LLaMa) and Falcon LLM provide contemporary examples for creating such localized hospital-based GPT applications [69, 70]. However, we foresee a few challenges of locally-run LLMs, such as potentially their suboptimal performance or the requirement for specialized hardware, as seen with GPT-3 [71]. Future research could therefore focus on developing smaller and more efficient LLMs that can run on everyday devices, similar to how Apple stores FaceID data locally on a chip [72].

Furthermore, LLMs were found to generate inaccurate or “hallucinated” information/citations [73]. To address this, human-labeled data and domain-specific GPT models will be necessary to further test, including Google’s Med-PaLM along with other emerging LLMs like perplexity.ai and Bing, which automatically provide references and links. Further research should also be conducted with the ChatGPT Plus version, which is connected to the internet, with explicit instruction to provide real references and links to those references.

Overall, recent guidelines made to address the logistical and ethical concerns of medical LLMs include the draft guidance issued by the FDA on predetermined change control plans for artificial intelligence/machine learning models [74]. Such guidelines may ultimately serve as frameworks for other researchers to further investigate addressing safety and regulation concerns related to LLMs in healthcare. On this note, as Comrie et al. propose, it may be worthwhile to investigate whether LLMs can be used to shape policies regarding their own regulation and integration, as well as the feasibility of hosting them locally [48].

But who should be involved in these discussions in the first place? Multiple of our extracted articles highlighted the importance of consulting various stakeholders for the successful implementation of LLMs in healthcare [16, 20]. Key stakeholders include clinical informaticians, developers, healthcare providers, policymakers, industry representatives, and civil society as a whole [75].

One barrier to having such discussions, as well as the successful implementation of LLMs into clinic spaces, was recently described by Lambert et al. [76]. Their article mentions that healthcare workers (HCWs) are hesitant to use AI in their practice due to several factors, including a justified concern of loss of work and/or professional autonomy, difficulty integrating AI like LLMs into the already established clinical operations, and potential loss of patient interactions [76]. However, the authors also report that improving training on using AI and involving all HCWs in the early stages of integration and creation of infrastructure to support the technology can ensure that HCWs are constantly being supported and heard during the process of bringing LLMs to healthcare settings.

### Objective 4: What could be a standardized evaluation framework for assessing LLMs’ clinical utility for future research studies?

The evaluation of text-based generation from large language models (LLMs) poses significant challenges due to the absence of a standardized evaluation framework. This is primarily because LLMs can produce multiple, variable responses and are highly dependent on their prompts. In this vein, majority of our quality ratings were designated as “N/A” given the recently emerging techniques for investigating and evaluating LLMs in medicine. In consideration of this, our review aimed to propose a more rigorous research design and present two main areas where such improvements can be made: in the study methods, and in how studies report their results.

i) **Disease/condition selection and contextual information**: To provide a comprehensive evaluation, we recommend researchers include a brief description of the epidemiology of the presented disease/condition in their articles. This description would cover aspects such as prevalence, severity, and acute nature, as our extracted articles have demonstrated how LLMs’ diagnostic accuracy vary depending on the severity of a patient’s symptom presentation. It would thus be important to further explore and monitor this relationship between accuracy and the severity of presentation (such as by using the estimated severity index, a five-level emergency department triage algorithm), considering that real-life patients present with a wide range of severity levels.
ii) **Exact wording and phrasing**: Based on our extracted articles, we recommend authors continue both recording and reporting the exact phrasing used when interacting with ChatGPT is paramount. This is because subtle differences in wording can lead to significantly varied responses with LLMs, even when the underlying intent is the same [37, 4, 20, 29–63]. This reporting practice will enable future studies to build upon or modify previous phrasings and vignette structures, and further test the replicability of LLMs’ responses to the same clinical prompts.
iii) **Inclusion of multiple question types**: To expand the application of research on LLMs, researchers could also incorporate both open-ended and closed-ended question formats in their evaluation. More specifically, this could include open-ended questions (similar to a real clinical setting), along with select-all-that-apply (SATA) questions and multiple-choice questions (MCQs) that are more relevant to medical education prompts. Furthermore, with all questions, it would be helpful to prompt LLMs to provide justifications for their responses [77]. Such prompting strategies may ultimately highlight the “concordance” or alignment of ChatGPT’s justifications with its answers. These questions may also elucidate the level of “uncertainty” by LLMs in their responses, what aspects of the prompt and patient scenario are difficult to ascertain, and ultimately for the users to understand what limitations exist in the LLMs’ responses [48]. An example follow-up prompt to incorporate all these points may be, “Based on the patient’s vignette, tell me what you think is the top differential diagnosis and explain your reasoning. Then, consider other differential diagnoses/management steps for this patient and why they are less likely/less appropriate in this clinical context.”
iv) **Research with one-shot or few-shot learning rather than zero-shot learning**: In order to determine what constitutes a “correct” diagnosis and ensure the accuracy of results from ChatGPT, it is beneficial to employ one-shot or few-shot learning techniques rather than zero-shot learning. More specifically, zero-shot learning refers to asking LLMs to generate a response without any fine-tuning or templates, while one-shot and few-shot learning refers to the generation of text in a specific style based on one or more templates provided, respectively. This can be done by conducting research where LLMs are provided with an accurate and comprehensive answer to follow, such as when asking LLMs to create EMR notes or providing a treatment plan. Furthermore, future research could also explore how LLMs adjust their responses after being given feedback about the accuracy, readability, and relevance of their responses to assess their ability to learn and improve within one chat session.
v) **Reporting results using standardized metrics for text-based output**: The evaluation of text-based generation from language models (LLMs) currently lacks a standardized approach, as there can be multiple valid or readable responses. Thus, beyond including human evaluators, we propose future research around LLMs’ clinical utility include the use of healthcare-specific evaluation methods for natural language generation (NLG) tasks like with LLMs. There is even very recently ongoing research on the alignment of agentic AI systems that do not require humans-in-the-loop (e.g. grants were released in December 2023 by OpenAI to fund research on superhuman AI systems that will not necessarily rely on human oversight at every step of the model’s decision-making process but is rather confirmed by a “superhuman” LLM that evaluate other LLM outputs in a manner similar to human reinforcement [78]. This may include using one or more of [21, 24, 79]:

1. Specification of the prompting parameters, including the temperature, model, seed version, max number of tokens permitted, frequency and presence penalties used to complete the study;
2. BLEU (Bilingual Evaluation Understudy) score to evaluate the accuracy of text that was translated by LLMs into another language;
3. ROUGE (Recall-Oriented Understudy for Gisting Evaluation) score that evaluates the quality of summaries created by text-generators;
4. METEOR (Metric for Evaluation of Translation with Explicit ORdering) that is used to evaluate automatic summarization created from natural language processing that compares an automatically produced summary against a reference produced by humans.
5. Perplexity, a metric used to examine the degree a language model can predict the distribution of a given text and its predicted words;
6. G-Eval, a novel evaluation technique created by Microsoft Cognitive Services Research Centre that has so far outperformed existing NLG evaluators in terms of correlation with human evaluations.
7. To curate a benchmark for LLMs in the medical domain, researchers can follow the example of MultiMedQA, a benchmark combining six existing medical question answering datasets spanning professional medicine, research and consumer queries and a new dataset of medical questions searched online, HealthSearchQA. The benchmark includes multiple-choice questions and a human evaluation framework for model answers along multiple axes including factuality, comprehension, reasoning, possible harm, and bias. The model was evaluated against multiple criteria, including scientific consensus, medical reasoning, knowledge recall, bias, and likelihood of possible harm.
8. Finally, a calculation of entropy may also be warranted. Entropy is a measure of the randomness or unpredictability of information, and specifically for LLM-generated text, entropy can be used to quantify the uncertainty of the next character or word in a sequence.

### Objective 5: What are some evidence gaps within the current literature and what are some potential future research directions for LLMs’ clinical applications?

The integration of language models, such as ChatGPT, into telemedicine outreach initiatives can significantly enhance healthcare accessibility and outreach to underserved populations, including in geographically isolated regions of the world and in developing countries. In this manner, it will also be crucial to consider the impact on high-income, middle-income, and low-income countries in terms of accessibility and costs. Conducting research in these diverse contexts will shed light on the feasibility, affordability, and potential benefits of implementing LLMs. For instance, prompting an LLM to consider local circumstances and available medical resources may yield particularly valuable insights in regions where there are such limited resources and options for patients. Furthermore, appropriately utilizing LLMs may lead to more consistent, standardized care across regions and ultimately promote consistent and equitable healthcare delivery.

Another significant area for investigation is the incorporation of social determinants of health (SDOH) and real, anonymized, consented patient cases to add complexity to LLM assessments [20, 30, 40]. While there is an abundance of research utilizing USMLE standardized, textbook-style questions, there is a scarcity of studies that leverage real patient data, which has been recognized as a limitation in several papers [20, 30, 40]. By including social factors such as socioeconomic status (SES), race, and sexuality, biases in the answers generated by LLMs can be directly assessed. It is crucial to address the inherent biases in LLMs, including mimicking extremist internet language, practical biases resulting from the underrepresentation of marginalized populations in data and research, and implicit biases introduced during fine-tuning by healthcare professionals [73].

The inability of language models (LLMs) to provide tailored recommendations based on regional guidelines and specific cut-off values for management, as highlighted by Yeo et al. (2023), is an important limitation that warrants further investigation [24]. Guidelines are typically developed based on trends derived from review methodologies, thus such reviews should be explicitly integrated and tested with LLMs. One challenge in this realm, however, is that certain medical conditions currently lack standardized guidelines [80, 81]. For example, conditions like reactive infectious mucocutaneous eruption, a rare but serious pediatric dermatological condition, currently have no established guidelines [80]. In such cases, LLMs, like ChatGPT, could potentially play a role in the creation of clinical guidelines. More specifically, LLMs may make the process more efficient by firstly confirming insights extracted by human experts, and subsequently offering novel insights and connections that may not have been discovered as quickly given the vast amount of clinical data and information synthesized. This capability opens up exciting possibilities for advancing medical knowledge and improving patient care through integrating LLMs into the guideline creation process. By leveraging their ability to find and generate connections and patterns in optimal patient care, LLMs can overall complement existing review methodologies used for the creation of clinical guidelines. Further research will need to be conducted to verify LLMs’ capabilities in assisting with such processes, especially given the potential impact on elevating or harming policies for population health.

Overall, given LLMs’ limitations, such as their tendency to hallucinate, the randomness of responses, and restrictions on a model’s training dataset (i.e. the “garbage-in garbage-out” phenomenon), general purpose LLMs are not in their current forms sufficient for medical decision-making purposes. Rather, they possess most potential in supporting patient navigation and EMR-related tasks, particularly given their ability to provide personalized responses to given prompts and summarize information efficiently and in an organized manner.

### Strengths and limitations

This scoping review used rigorous and transparent methods. Its conduct was guided by the PRISMA-ScR reporting checklist, as well as a prospectively published protocol designed by expert reviewers with knowledge in evidence synthesis and scoping reviews. Structured, librarian-assisted database searches were conducted in three major literature databases coupled with extensive handsearching, which ensured comprehensive inclusion of relevant articles. Additionally, each record retrieved from databases were reviewed in-duplicate using an established review platform (Covidence) to ensure that all citations and articles were properly tracked and accounted for during the screening process.

Despite our attempts to conduct a comprehensive search, this review may not have identified all available studies. Our search strategy included 8 key terms to cover the concept of LLM; however, other terms may exist, especially since a diverse set of LLM naming schemes are actively being used. Additionally, our strategy used terms relating to clinical practice to filter out potentially irrelevant records. This may inadvertently exclude relevant articles that did not have health-related terms in its title, keywords, or MeSH entries. Finally, much has evolved in the world of LLMs since we began our study and completed our search; subsequently, while we have strived to incorporate several updates into our discussions, a limitation is that our main analysis is mainly based on the studies that were available at the time that we performed our search and analyses.

As this was a scoping review, we did not assess the risks of bias of the included studies. There is also currently no validated quality assessment tool available for assessing the type of exploratory machine learning studies included in this review, as noted. We are also unable to verify the accuracy and validity of findings reported in our included preprint articles, as they have not undergone peer-review.

## Conclusion

Large language models (LLMs), particularly ChatGPT, have shown promise in various clinical applications, ranging from the creation of patient notes to helping healthcare providers diagnose rare conditions. However, it is important to recognize the inherent limitations of artificial intelligence (AI) systems. Overall, as our extracted articles also reinforce, there is a place for humans-in-the-loop to oversee LLMs utility in clinical settings to ensure erroneous recommendations or inadequate diagnoses do not cause patient harm. The responsibility to ensure the accuracy and reliability of LLMs before integration into clinical settings further rests on multiple stakeholders - particularly researchers. Overall, validation and replication studies are essential, and our paper synthesizes several areas of ongoing logistical and ethicolegal concerns. We also propose standardized techniques that can be integrated into future research to better address the challenges and uncertainties associated with LLMs in medicine. All in all, LLMs hold significant potential, and the continued exploration and careful navigation of these challenges will be crucial to understand their benefits and drawbacks in healthcare settings.

### Electronic supplementary material

Below is the link to the electronic supplementary material.

Supplementary Material 1

## Data Availability

All data and materials available upon request.
