# Supplementary material for: Assessing the research landscape and clinical utility of large language models: a scoping review
Source: BMC Med Inform Decis Mak. 2024 Mar 12;24:72. doi: 10.1186/s12911-024-02459-6 (PMC10936025; doi:10.1186/s12911-024-02459-6)
Supplement: Supplementary file 1 — Supplementary Material 1 [file 12911_2024_2459_MOESM1_ESM.docx]

[Table 1. Summary of studies discussing LLMs’ utility in compiling patient notes in the EMR system or in creating case reports. 1](#_lc9j54ugnpya)

[Table 2. Summary of studies discussing ethical, logistical, and legal implications of LLMs in the clinical setting. 4](#_8lydljpfaldb)

[Table 3. Summary of studies discussing LLMs’ utility in supporting patients in navigating the healthcare system. 11](#_qg31ab59s37k)

[Table 4. Summary of studies discussing LLMs’ utility in clinical decision-making processes (i.e. management, risk prediction, diagnostic support). 18](#_vkl5xvemvwwd)

# NR = not reported

N/A = not applicable

# Table 1. Summary of studies discussing LLMs’ utility in compiling patient notes in the EMR system or in creating case reports.

| **Authors and Date of publication** | **Date published (month/year)** | **Title** | **Type of study or article and journal name** | **Study aims** | **Study Methods (if applicable)** | **Study Results** | **Suggested areas of further research** | **Quality of Evidence** Ratings:  1 - Properly powered and conducted RCT; systematic review with meta-analysis  2 - Well-designed controlled trial without randomization; prospective comparative cohort trial  3 - Case-control studies; retrospective cohort study  4 - Case series with or without intervention; cross-sectional study  5 - Opinion of respected authorities; case reports |
| --- | --- | --- | --- | --- | --- | --- | --- | --- |
| Ali et al. | April 2023 | Using ChatGPT to write patient clinic letters | Research Letter; Lancet | To investigate if ChatGPT can improve the efficiency, accuracy, humanness, and readability of patient letters | Instructed ChatGPT to generate clinical letters for hypothetical skin cancer cases. An online tool was used to judge the readability, with accuracy & humanness being rated by two clinicians. | Letter readability was at 9th grade level. Accuracy and humanness differed significantly based on cancer type. | Investigate the use of voice-to-text recognition software with ChatGPT. Future studies should use more medically focused prompts, and test letters for different languages & cultures. | 5 |
| Cascella et al. 2023 | Feb 2023 | Evaluating the Feasibility of ChatGPT in Healthcare: An Analysis of Multiple Clinical and Research Scenarios | Brief Report; J Med Syst. | To test the feasibility of ChatGPT in providing clinical decision support and assisting with medical research, identifying areas of misuse, and reason about public health topics. | 1) Prompted ChatGPT to create structured ICU notes. Prompt included info about treatment, lab values, etc. in random order.  2) Prompted ChatGPT to describe situations of misuse in medicine.  3) Prompted ChatGPT to discuss definitions of seniority in public health | ChatGPT correctly organized misplaced data when creating notes. However, it lacked expertise at times, and exhibited hallucination. Additionally, ChatGPT perpetuated stereotypes about seniority. | ChatGPT has the potential to handle complex info via EHRs and clinical notes. Hallucination and bias of ChatGPT should be further studied. | 5 |
| Sajan & Kyle 2023 | Feb 2023 | ChatGPT: the future of discharge summaries? | Commentary; Lancet | To comment on the potential and limitations of ChatGPT in creating discharge summaries. | N/A | ChatGPT can rapidly answer questions in a conversational manner. It can keep track of previous responses. It can potentially be used to automate creation of standardized discharge summaries. However, quality of output may depend on the dataset. Other concerns include privacy and tech failures (requiring manual checks by healthcare professionals) | Pilot studies needed to gather perspectives from patients, staff, and other stakeholders about the use of ChatGPT in patient discharge summaries or other aspects of healthcare. | 5 |
| Lee et al. 2023 | March 2023 | Benefits, Limits, and Risks of GPT-4 as an AI Chatbot for Medicine | Brief Report; N Engl J Med | To investigate the use of GPT-4 in medical note writing and consultation. | Provided GPT-4 with a transcript of a patient-physician conversation and asked it to write a note about the visit. This note was then assessed. | GPT-4 can identify errors and correct medical notes. It can answer medical test questions with reasoning. It can help with validation of created medical notes. Can potentially improve patient outcomes and increase efficiency.  However, GPT-4 is not trained on EHR data. Oftentimes responses are influenced by the user. Have a tendency to hallucinate. | N/A | 5 |
| Puthenpura et al. | March 2023 | Personality Changes and Staring Spells in a 12-Year-Old Child: A Case Report Incorporating ChatGPT, a Natural Language Processing Tool Driven By Artificial Intelligence | Case report; Cureus | To report a patient case of personality changes and subsequent diagnosis of an intracranial lesion using Chat-GPT. | All pertinent information about the patient was constructed and added to ChatGPT. Output from ChatGPT was reviewed for its accuracy. An abstract was created using ChatGPT as well. All prompts and outputs were reported in the appendix sections. | Benefits included streamlining the process and providing a general structure that aligns with the themes of the paper. However, information was sometimes incomplete or difficult to understand without expert insights. References were sometimes non-existent. | NR | 5 |
| Lantz, 2023 | March 2023 | Toxic Epidermal Necrolysis in a Critically Ill African American Woman: A Case Report Written With ChatGPT Assistance. | Case report; Cureus | To describe the diagnosis and management of a patient with TEN with the support of Chat-GPT, and to discuss the benefits and drawbacks of using Chat-GPT in writing case reports. | Used Chat-GPT to generate text responses on TEN’s disease descriptions, drug interactions, and references. All information was fact-checked with the literature; 4 out of 7 references were fake, most real references were from 10+ years ago and therefore less relevant. | Benefits included ChatGPT’s efficiency and saved time. However, cons included that many references were non-existent, and all information needed to be fact-checked anyways by the authors. | NR | 5 |

# Table 2. Summary of studies discussing ethical, logistical, and legal implications of LLMs in the clinical setting.

| **Authors** | **Date published (month/year)** | **Title** | **Type of study or article** | **Study aims** | **Study Methods (if applicable)** | **Study Results** | **Suggested areas of further research** | **Quality of Evidence** Ratings:  1 - Properly powered and conducted RCT; systematic review with meta-analysis  2 - Well-designed controlled trial without randomization; prospective comparative cohort trial  3 - Case-control studies; retrospective cohort study  4 - Case series with or without intervention; cross-sectional study  5 - Opinion of respected authorities; case reports |
| --- | --- | --- | --- | --- | --- | --- | --- | --- |
| Beltrami et al. 2023 | March 2023 | Consulting ChatGPT: Ethical dilemmas in language model artificial intelligence. | Commentary ; J Am Acad Dermatol. | To clarify whether it is ethical and reasonable to use ChatGPT in clinical decision supports based on the pillars of beneficence, nonmaleficence, physician integrity, justice. | N/A | Benefits include that ChatGPT  provides medically correct interpretations of specific clinical cases, improving accuracy and physician competence. Offers breadth of knowledge to users, reduces human error, and enhances decision-making in dermatology. Free and accessible.  Cons include that ChatGPT may provide false, biased, or outdated information based on its source data, may lack awareness of current guidelines. Over-reliance on ChatGPT without critical reasoning can cause harm to patients. Patients may seek expertise of ChatGPT instead of a physician, leading to potential harm and lost trust. | Discussion and integration of physician oversight over ChatGPT's integration into healthcare setting, particularly in communities that experience difficulty in accessing physicians like dermatologists and may therefore see ChatGPT’s integration into its healthcare system in the near future. | 5 |
| Sezgin et al. 2022 | March 2023 | Operationalizing and Implementing Pretrained, Large Artificial Intelligence Linguistic Models in the US Health Care System: Outlook of Generative Pretrained Transformer 3 (GPT-3) as a Service Model | Brief report ; JMIR Med Inform. | To discuss how GPT3 can be implemented in clinical practice, including what infrastructure can be used to integrate GPT3 in clinical practice, what costs and model biases are involved, how to evaluate model performance, and how to remain HIPAA compliant. | N/A | GPT3 requires more specialized hardware for execution given its high processing needs.  Possesses inherent biases due to mimicking extremist internet language, practical biases due to under integration of marginalized populations in data and research, implicit biases of healthcare professionals during fine-tuning.  Currently no standardized way to evaluate text-based generation, as multiple, variable responses from LLMs can be considered “correct” or “readable”.  HIPAA compliances will take time - Amazon’s Alexa for instance took 5 years before it was recognized as HIPAA compliant and therefore added to healthcare delivery modalities | Recommendation to run GPT-3 as a service (i.e. service request to GPT-3 service that will then process and submit the request). Integration of GPT-3 with cloud platforms may reduce operating burden but will be very expensive.  Future directions include active bias testing with clear procedures and “human-in-the-loop”  Healthcare-specific evaluation methods for text generation tasks required (i.e. similarly to Bilingual Evaluation Understudy BLEU or Metric for Evaluation of Translation with Explicit Ordering METEOR)  HIPAA compliance will take even longer given that GPT-3 is a “black-box” model.  Require clarity on how exactly patients’ data will or will not be used to ensure trust. | 5 |
| Xue et al. 2023 | Feb 2023 | The potential impact of ChatGPT in clinical and translational medicine | Commentary; Clin Transl Med. | To discuss ChatGPT’s applications in mental health, epidemiological research, and medical writing, the ethical concerns surrounding its use, and its potential benefits in medicine. | N/A | Benefits include the potential for an objective and evidence-based approach to decision-making, assist in disease detection and prognosis prediction, and improved patient outcomes using ChatGPT.  Cons include the potential negative impacts of AI and ChatGPT, privacy concerns, bias, and discrimination. | ChatGPT is limited in specific medical-related conversations, such as accurately judging infections or other causes of common symptoms such as fever.  ChatGPT does not update training data in real-time and cannot independently handle the complex work of clinical practice in its current version.  Suggests that further research and development are needed to ensure its responsible use. | 5 |
| Sallam 2023 | Preprint published in Feb 2023; published March 2023 | The Utility of ChatGPT as an Example of Large Language Models in Healthcare Education, Research and Practice: Systematic Review on the Future Perspectives and Potential Limitations | Preprint research article; Healthcare (Basel) | To review the future perspectives of ChatGPT as a prime example of LLMs in healthcare education, academic/scientific writing, and healthcare practice based on existing evidence, as well as identify potential limitations and concerns associated with the application of ChatGPT in these areas. | The review analyzed eligible scientific research or preprints addressing ChatGPT in healthcare practice/research, healthcare education, and academic writing. Exclusion criteria included non-English records, records addressing ChatGPT in subjects outside the eligibility criteria, and articles from non-academic sources. The information sources included PubMed/MEDLINE and Google Scholar. The review identified a total of 82 relevant records. | Benefits include the utility in analyzing massive datasets efficiently, code generation, drug discovery and development, cost-saving, documentation, personalized medicine, and improved health literacy.  Cons include ethical issues, including the risk of bias, plagiarism, copyright issues, transparency issues, legal issues, lack of originality, incorrect responses, limited knowledge, and inaccurate citations. | Ethical concerns, transparency, and legal issues should be considered carefully. Additionally research and appropriate guidelines and regulations are urgently needed for the use of ChatGPT in medicine. | 1 |
| Haupt & Marks 2023 | March 2023 | AI-Generated Medical Advice-GPT and Beyond. | Brief report ; JAMA Network | To discuss the legal boundaries and implications of GPT in healthcare. | N/A | Like with smartphones, EMRs, desk references - GPT could be regulated as a routine support system that provide clinical support. Liability could still be assigned to professionals and providers who use GPT given that, like with other clinical supports, the information was first being filtered through the professional and their judgment call therefore the provider is responsible for the outcome.  Consumer-facing products (i.e. Twitter, Google, etc.) do not need to follow HIPAA, privacy rules also do not apply. The same could happen if Chat-GPT is used in consumer products (e.g. 2023 Koko) | Legally nebulous areas include the use of GPT when AI becomes the de facto for providing medical advice (such as in basic mental health care to patients, or replacing staff who perform triage roles) as the tool is now substituting human judgment rather than augmenting it. | 5 |

# Table 3. Summary of studies discussing LLMs’ utility in supporting patients in navigating the healthcare system.

| **Authors** | **Date published (month/year)** | **Title** | **Type of study or article** | **Study aims** | **Study Methods (if applicable)** | **Study Results** | **Suggested areas of further research** | **Quality of Evidence** Ratings:  1 - Properly powered and conducted RCT; systematic review with meta-analysis  2 - Well-designed controlled trial without randomization; prospective comparative cohort trial  3 - Case-control studies; retrospective cohort study  4 - Case series with or without intervention; cross-sectional study  5 - Opinion of respected authorities; case reports |
| --- | --- | --- | --- | --- | --- | --- | --- | --- |
| Yeo et al. 2023 | Feb 2023 | Assessing the performance of ChatGPT in answering questions regarding cirrhosis and hepatocellular carcinoma | Research Article; Clin Mol Hepatol. | To assess the accuracy and reproducibility of ChatGPT in answering questions regarding knowledge, management, and emotional support for cirrhosis and hepatocellular carcinoma. | Questions about cirrhosis or hepatocellular carcinoma entered as independent prompts. Questions from professional societies, institutions, and patient support groups via Facebook posts.  Review and grading of each response done by two transplant hepatologist reviewers using a 4 point system.  Reproducibility determined by assessing the similarity of two responses to each individual question. | Benefits include ChatGPT provided extensive knowledge of cirrhosis and HCC with high accuracy. Performance better in basic knowledge, lifestyle, and treatment than in diagnosis and preventive medicine  However, only small proportions of answers were labeled as comprehensive. Unable to provide tailored recommendations based on regional guidelines and specific cut-off values for management. | Future studies needed to examine the utility of ChatGPT in patient education and monitor accuracy and reproducibility of its responses to patient questions.  Studies on how ChatGPT can draft a framework for each question asked by patients and caregivers, thereby increasing efficiency for providers.  Studies on how ChatGPT can be programmed to prompt clarifications from users to fine-tune the questions and provide responses with higher accuracy. | N/A |
| Lyu et al., 2023 | Pre-print published in May 2023; Article published Dec 2023 | Translating Radiology Reports into Plain Language using ChatGPT and GPT-4 with Prompt Learning: Promising Results, Limitations, and Potential | Pre-print research article; Vis Comput Ind Biomed Art. | To evaluate the performance of ChatGPT in translating radiology reports into layman versions and to assess the quality of the suggestions provided by ChatGPT for both patients and healthcare providers. | The study collected chest CT screening reports and brain MRI screening reports. ChatGPT was given three prompts: to translate a radiology report into plain language, to provide suggestions for the patient, and to provide suggestions for the healthcare provider. Responses were evaluated by two experienced radiologists evaluated the quality of the ChatGPT responses, focusing on overall score, completeness, and correctness. | ChatGPT can generate improved results with clearer and more specific instructions on which information should be kept in a radiology report. The study found that ChatGPT's performances were similar with multiple semantically similar prompts when there were no clear instructions on how to preserve information.  However, ChatGPT's translations were not unique for any given radiology report, with different lengths of reorganized paragraphs and flexible choices of alternative words. There was a distinctive response from ChatGPT despite the same prompt and radiology report provided. ChatGPT tended to generate over-simplified translations and left out important information. | NR | N/A |
| Ali et al., 2023 | May 2023 | Bridging the Literacy Gap for Surgical Consents: An AI-Human Expert Collaborative Approach | Pre-print (still a pre-print as of Dec 2023) | To investigate the use of GPT-4’s in health literacy, namely in simplifying surgical consent forms and evaluate its potential based on improved form readability and specificity of the forms, and via validation of content appropriateness | Consent forms were from across several institutions. Pre- and post-simplification readability metrics after GPT-4’s use were compared and evaluated through a subspecialty surgeon panel. | Significantly improved readability from college level to grade 8 level, improved reading ease, active voice used rather than passive voice, medical jargon was replaced with more accessible terms. Final independent human review helped verify against “hallucinations” or other inaccuracies. Rubric criteria also helped to ensure clinical accuracy was not sacrificed. | Such simplification of crucial but complex forms could be applied to other jurisdictions, such as in law (e.g. malpractice trials). | N/A |
| Zhu et al. 2023 | April 2023 | Can the ChatGPT and other Large Language Models with internet-connected database solve the questions and concerns of patient with prostate cancer? | Research Letter; J. Transl. Med. | To evaluate whether LLMs can be used as a consultant for prostate cancer patients, whether LLMs can answer questions associated with the latest developments in prostate cancer management, whether LLMs with internet-connected databases could provide more up-to-date information compared with ChatGPT. | Sought to assess the accuracy, comprehensiveness, patient readability, and inclusion of humanistic care for the patient in the answers provided by LLMs.  Performance of different large language models (LLMs) including ChatGPT (both free and paid version), YouChat, NeevaAI, Perplexity (concise and detailed model), and Chatsonic, in answering common questions related to prostate cancer and providing humanistic care for patients was evaluated. | LLMs could provide correct answers to most questions, and the accuracy of most LLMs' responses was above 90%. | Suggests that using LLMs for future doctor-patient communication in healthcare is feasible and that it sheds light upon the possible future of AI-based healthcare. Current LLMs sometimes use outdated information or provide the wrong information but the authors expect LLMs to get better with time. | 5 |
| Cox et al. 2023 | April 2023 | Utilizing ChatGPT-4 for Providing Medical Information on Blepharoplasties to Patients | Research letter; Aesthetic Surg J. | To determine when ChatGPT-4 can be used to bridge communication gaps between providers and patients. | Two prompts were provided to ChatGPT-4; the first question around what are the risks and complications associated with blepharoplasty, while the second is how the patient can expect their eyes to look over time. | Answers from ChatGPT-4 were safe, quick, and appropriate for patients without excessive jargon included. | Recommended updates past 2021 data. Quality control measures needed to validate and ensure precision of the information before its dissemination to patients. Integration into EMRs can help streamline patient education. | 5 |
| Suresh et al. 2023 | May 2023 | Utility of GPT-4 as an Informational Patient Resource in Otolaryngology | Preprint; still a pre-print as of Dec 2023 | Evaluates GPT-4 responses to questions based on the American Academy of Otolaryngology’s guidelines from patients’ perspective. | ChatGPT was given 18 queries to answer. Clinicians evaluated responses based on safety, accuracy, and comprehensiveness, from patients’ perspective. | All responses were safe. 14/18 (78%) responses were accurate, and 15/18 (83%) were comprehensive. | Domain-specific models trained on verified corpus of biomedical literature should be explored to increase accuracy. | N/A |

# Table 4. Summary of studies discussing LLMs’ utility in clinical decision-making processes (i.e. management, risk prediction, diagnostic support).

| **Authors** | **Date published (month/year)** | **Title** | **Type of study or article** | **Study aims** | **Study Methods (if applicable)** | **Study Results** | **Suggested areas of further research** | **Quality of Evidence** Ratings:  1 - Properly powered and conducted RCT; systematic review with meta-analysis  2 - Well-designed controlled trial without randomization; prospective comparative cohort trial  3 - Case-control studies; retrospective cohort study  4 - Case series with or without intervention; cross-sectional study  5 - Opinion of respected authorities; case reports |
| --- | --- | --- | --- | --- | --- | --- | --- | --- |
| Chari et al. 2023 | Feb 2023 | Informing clinical assessment by contextualizing post-hoc explanations of risk prediction models in type-2 diabetes | Research article; ACM Digital Library | To investigate several LLMs' (including BERT’s) potential in comorbidity risk predictions based on a patient's clinical state, risk fo complications, and algorithm performance supporting the predictions for type 2 diabetes and CKD | Prompted LLMs to generate answers for questions related to providing a summary for patients, risk predictions, contributing factors, and lab interpretations for T2DM. Same questions were asked and responses collected from an expert medical panel. | Pros include pre-filtering answer sets and narrowing questions can help make the output more precise (ex: in relation to medication question).  Cons include unsupervised adaptations of BERT could only reach a maximum accuracy, required domain specific adaptations to incorporate medical guidelines. | Investigate the integration of sources like systematic reviews, RCTs, cohort studies, expert opinions to improve the evidence pyramid aspect of BERT models. | N/A |
| Baumgartner 2023 | Feb 2023 | The opportunities and pitfalls of ChatGPT in clinical and translational medicine | Commentary ; Sec. Healthcare Professions Education | To explore the benefits, challenges, pitfalls, and future perspectives of ChatGPT in clinical and translational medicine through the lens of ChatGPT itself | N/A | Pros include access to up-to-date information in real-time, improved patient engagement and tracking health information, reduced workload in administrative tasks.  Cons include data privacy concerns due to access to personal health information, inconsistent accuracy for complex medical questions, bias in the training dataset that perpetuates potential harms, misleading information, and dependence on technology if used as a crutch for healthcare providers, different responses each time same question is asked - not standardized. | As dataset for ChatGPT expands, so too will its accuracy. Integration of ChatGPT with electronic health records should also help improve transition of information between healthcare providers and patients. Personalized medicine through tailored medical advice based on individual patient data and health histories. Further areas of application include: data-driven medical research, decision supports, imaging analysis for diagnosis, pattern recognition on ECGs, EEFs, EMGs. | 5 |
| DiGiorgio et al. 2023 | March 2023 | Artificial Intelligence in Medicine & ChatGPT: De-Tether the Physician. | Brief report; J Med Syst. | To shed light on how LLMs like ChatGPT can be integrated into the medical field and what types of tasks ChatGPT can provide support on, further considering regulatory guidelines | N/A | Recommendations:  AI systems should seamlessly integrate with physicians' clinical decision-making process, providing suggestions rather than questioning their decisions. They can assist with tasks like filling out orders, sending codes, and integrating notes into the electronic health record (EHR). Physicians can then review and revise the AI-generated notes. AI can also help improve billing processes and track quality metrics.  Pros include that AI can help physicians pass medical exams like the USMLE without extensive training, potentially reducing medical errors and improving the quality of care. It is well-suited for "textbook patients" and addressing specific clinical questions with narrow scopes.  Cons include that AI may provide generic or "canned responses" when faced with real-world symptoms and prompts. It cannot replace human judgment in diagnosing and treating diseases. Its probabilistic algorithms are limited in their understanding and may not capture the complexities of real patients or account for nuances in body language, tone, family dynamics, and patient priorities. Flawed algorithms based on faulty data or biased studies can perpetuate harm, especially if they fail to incorporate social determinants of health (SDOH) that are increasingly important in healthcare literature. | AI should be used to help "unburden" physicians and allow physicians to focus on their relationship with their patients (and therefore "untethered" from the computer). Regulations should allow for innovated AI to be integrated into the real-world but in an equitable and fair manner (ground-up rather than top-down approach). Ultimately, integration of AI for improving administrative tasks will likely require less oversight in comparison to integrating AI for treatments or diagnoses. | 5 |
| Khan et al. 2023 | March 2023 | ChatGPT-Reshaping medical education and clinical management. | Research letter; Pak J Med Sci. | To comment on areas where ChatGPT can be a tool in medical education, research, and clinical settings while describing its limitations. | N/A | ChatGPT has several potential applications in healthcare, such as language translation to overcome cultural and linguistic barriers with patients, improving documentation accuracy and efficiency, serving as a decision support tool for healthcare professionals, and facilitating communication with patients for queries and appointment management.  Pros include improved documentation, decision support, and communication with patients, even across different languages. Helps reduce human error and optimize time in clinical documentation and administrative tasks.  Cons include ChatGPT's knowledge is limited to data available up until 2021, potentially resulting in outdated information. There is a risk of generating irrelevant or incorrect information due to the lack of recent data. | ChatGPT cannot replace human thought, however, its rise in use and impact are inevitable therefore need to adopt to those changes with an open mind for the benefits of clinic settings and also in other realms, including medical education or research. | 5 |
| Rao et al. 2023 | Feb 2023; published Oct 2023 | Evaluating ChatGPT as an Adjunct for Radiologic Decision-Making | Pre-print research article; JACR | To investigate ChatGPT's ability to provide clinical decision support in radiology for identifying imaging services for breast cancer screening vs breast pain. | Compared ChatGPT's responses to prompts about breast pain and breast cancer screening with the American College of Radiology Appropriateness Criteria. Scoring based on whether the imaging modalities and suggestions were in line with the ACR guidelines. Prompt formats included open-ended format, select all that apply format. | ChatGPT showed moderate accuracy in radiologic decision-making, with higher accuracy in Select All That Apply (SATA) prompts compared to One-Best-Answer (OE) prompts. It performed better in breast cancer screening scenarios than in breast pain scenarios, and its accuracy increased with the severity of the clinical presentation for breast cancer screening but decreased for breast pain. ChatGPT had difficulty identifying cases of futile care and sometimes provided multiple recommendations instead of a single recommendation as requested. Despite these limitations,  ChatGPT demonstrated an overall accuracy of over 50% and provided extensive rationale for its recommendations that aligned with guidelines. Its best application in the clinical setting would likely be providing a list of options along with its reasoning. It showed high accuracy in breast cancer screening, making it useful for screening prompts and resource stewardship.  Shortcomings included that ChatGPT experienced misalignment, hallucinations, faulty errors, falsely attributing information, and providing more information than requested. | NR | N/A |
| Rao et al. 2023 | Feb 2023; officially published May 2, 2023 | Assessing the Utility of ChatGPT Throughout the Entire Clinical Workflow | Pre-print research article; JMIR | To explore ChatGPT's ability for clinical decision supports via performance on standardized clinical vignettes. | In this study, 36 clinical vignettes from the MSD Clinical Manual were used as input for ChatGPT. The vignettes covered common healthcare scenarios, including information on the patient's history of present illness, review of systems, physical exams, and laboratory test results. The conversations with ChatGPT were conducted in separate sessions to avoid cross-influence between vignettes but continued within each session for interactive learning. SATA type questions were used to simulate the requirement for differential diagnosis, diagnostic work-up, and clinical management decisions. To account for response variation, each vignette was tested three times by different users. Vignettes were rated for clinical acuity using the Emergency Severity Index (ESI). | Pros include ChatGPT may be one of the first models that can be used for any type of clinical support and throughout the entire clinical situation (rather than only a specific area of care, such as in triage, communication aids, etc.). ChatGPT contains a "memory" function from previous conversations, allows for reflection of iterative nature of clinical settings in Chat-GPT.  Cons include that ChatGPT is self-contained and therefore does not have the option to search the internet when generating responses. ChatGPT sometimes refuses to provide a diagnosis, indicating that it is not always able to navigate clinical situations where there is a well-established standard of care as well as in complex, ambiguous clinical scenarios. Highlights potential need for domain-specific GPTs. ChatGPT lacks actual reasoning skills because in the end, its answers are based on what is the most likely "token" to complete an answer rather than the actual meaning of the answer - sometimes ChatGPT thus recommends futile care or does not provide the appropriate diagnosis due to the model's technique. | To evaluate ChatGPT's ability to recommend appropriate utilization of resources by asking questions like, "what tests are appropriate clinically while taking cost management into account?" ChatGPT did not appear to have age or gender biases, however for other atypical presentations such as racial differences, this may lead to differences in accuracy therefore future investigation is needed in future studies. | N/A |
| Abdel-Messih et al. 2023 | March 2023 | ChatGPT in Clinical Toxicology | Research letter; JMIR Med Educ. | To explore the use of ChatGPT in clinical toxicology using a typical vignette. Mainly focused on the accuracy of the response and ability to answer questions. | Prompted ChatGPT with one short answer clinical vignette with multiple questions. Assessed the answer on quality and accuracy. | ChatGPT performs well in a common clinical toxicology case, offering a sound response and explanation of the reasoning behind the response. | NR | 5 |
| Ufuk 2023 | March 2023 | The Role and Limitations of Large Language Models Such as ChatGPT in Clinical Settings and Medical Journalism. | Research letter; RSNA | To provide comments and insights on the limitations and potential implications of large language models (LLMs) such as ChatGPT in clinical settings and medical journalism. The author discusses the importance of transparency and explainable AI techniques in fields such as healthcare and finance, and highlights the potential of LLMs in assisting radiologists in their workflow, but emphasizes the need for human verification of generated reports. | N/A | Importance of transparency and explainable AI techniques in fields such as healthcare and finance, and highlights the potential of LLMs in assisting radiologists in their workflow. There is a need for human verification of generated reports. Open data and open science publishing is needed for models to detect fabricated articles and promoting transparency and reproducibility in scientific research and clinical settings. | NR | 5 |
| Yang et al. 2022 | Feb 2022 | GatorTron: A Large Language Model for Clinical Natural Language Processing. | Research article; NPJ Digit. Med. | To create a large clinical transformer model called GatorTron by using a dataset of over 90 billion words, to scale up the GatorTron model by training it with different numbers of parameters, and compare its performance with existing biomedical and clinical transformers like BioBERT and ClinicalBERT. | Created and coded GatorTron. | GatorTron showed superior performance compared to existing biomedical and clinical transformers for all evaluated clinical NLP tasks, especially for complex tasks like natural language inference and medical question answering. Scaling up the size of the GatorTron model showed consistent improvements for most tasks, indicating the significance of larger transformer models for complex NLP tasks. | Stressed the importance of leveraging NLP powered by pretrained language models in medical AI systems using increased parameters and training data size to enhance NLP tasks in healthcare delivery. | N/A |
| Nori et al. 2023 | March 2023 | Capabilities of GPT-4 on Medical Challenge Problems | Research article; Microsoft | To explore the capabilities of large language models (LLMs) in medical problem solving, particularly in comparison to the recently released GPT-4 model and its predecessors in the GPT family. | To evaluate GPT-4 against its predecessor GPT 3.5 using six datasets that cover different aspects of medical knowledge and reasoning (from USMLE Sample Exam, USMLE Self Assessments, MedQA, PubMedQA, MedMCQA, and MMLU) | GPT-4 exhibits significant improvement over previous models in terms of performance compared to GPT-3.5. It also performs well in medical challenge benchmarks, including different languages.  Pros include the potential to aid precision clinical medicine by providing analytics, reminders, and decision support to healthcare practitioners. It can assist in formulating and revising differential diagnoses, identifying relevant tests, and developing therapy plans.  Cons include that the model must have robust standards for verifying the information generated by GPT-4 and similar models, ensuring adherence to high-quality standards. Addressing and mitigating biases in healthcare delivery and data is crucial to avoid potential biases in AI system outputs. Research is necessary to understand the fairness of healthcare recommendations generated by large-scale language models. | To explore richer prompting strategies to improve model performance, such as ensemble approaches and information retrieval tools, as well as discovering new prompting patterns that work optimally for GPT-4.  To leverage LLMs’ possibilities to provide information, communication, screening, and decision support in under-served regions and to raise the competency of physicians' assistants. | N/A |
| Chen et al. 2023 | March 2023 | The utility of ChatGPT for cancer treatment information | Pre-print research article; still a pre-print on Dec 2023 | To evaluate ChatGPT's performance to provide treatment recommendations for breast, prostate, and lung cancer | Four prompts were provided to ChatGPT and scored by oncologists based on concordance, particularly with guidelines, as well as reliability and robustness of responses. | ChatGPT provided at least one NCCN-concordant treatment in 98% of the prompts (102 out of 104), indicating a high level of alignment with the NCCN guidelines. However, ChatGPT also generated non-concordant treatments in 34.3% of the correct responses. Several of the outputs also did not offer any specific treatment recommendations, suggesting limitations in providing actionable guidance.  High disagreements among scorers indicated that the outputs of large language models like ChatGPT can be ambiguous and challenging to interpret/standardize. ChatGPT occasionally provided incorrect recommendations alongside correct ones, potentially introducing errors that are challenging to identify. | NR | N/A |
| Nastasi et al. 2023 | March 2023; published Oct 2023 | Does ChatGPT Provide Appropriate and Equitable Medical Advice?: A Vignette-Based, Clinical Evaluation Across Care Contexts | Pre-print research article; Nature Sci. Rep. | To assess ChatGPT's response to clinical questions, including prevention, management of acute illness, and end-of-life decision-making. Also assessed how the responses differed by patient race, gender, and insurance status. | Responses to the vignettes were assessed for clinical appropriateness, acknowledgement of uncertainty, appropriate follow-up reasoning, recommendation type, and differences by demographic characteristics. Outcomes were coded by two physicians and disagreements were resolved via consensus. | 3% of responses were clinically inappropriate. Recommendations in response to advice-seeking questions were completely absent (N=34, 35%), general (N=18, 18%), or specific (N=44, 46%). Fifty-three (55%) explicitly considered social factors like race or insurance status, which in some cases changed clinical recommendations.  Overall:  ChatGPT usually provided appropriate medical advice in response to advice-seeking questions. Responses lacked personalized nuance or follow-up questions that would be expected from a physician. Responses also did not provide recommendations that considered pain quality, duration, associated symptoms or contextual clinical factors when evaluating cases. Responses varied greatly depending on social factors, often without a clinical basis (e.g. sending uninsured patients to a community health clinic while sending insured patients to ED for the same presentation). The content and type of responses also varied widely and arbitrarily. | Future training on medical corpora, clinician-supervised feedback, and augmenting awareness of uncertainty and information seeking may improve medical advice provided by future LLMs. | N/A |
| Haemmerli et al. 2023 | March 2023 | ChatGPT in glioma patient adjuvant therapy decision making: ready to assume the role of a doctor in the tumour board? | Pre-print research paper; BMJ HCI | To evaluate ChatGPT’s recommendations for glioma management. | Five tumor board experts evaluated ChatGPT's output on a scale of 0 to 10, with 0 indicating complete disagreement between ChatGPT output and the original tumor board decisions, and 10 indicating complete agreement. Evaluated based on: diagnosis, the proposed treatments, the consideration of the patient's functional status to support adjuvant therapy, the proposed regimen of adjuvant therapy, and the overall accuracy of ChatGPT with respect to its answers.  Ten glioma cases were randomly chosen from a tumour registry.Specific prompts presented to ChatGPT include the main clinical information, admission context, preoperative radiological and surgical information, postoperative clinical information, neuropathological findings, and results of the immunohistochemical and molecular examination. Two questions were posed to ChatGPT: 1) What is the best adjuvant treatment? and 2) What would be the regimen of radiotherapy and chemotherapy for this patient? | Output from ChatGPT were rated as poor for diagnosis, good for treatment recommendation, good for therapy regimen, moderate for functional status consideration, and moderate for overall agreement with recommendations. ChatGPT's performance is mediocre due to its less extensive and not well-documented knowledge base. It is also likely that ChatGPT's protocol has limited its ability to provide medical advice. | Future studies could explore ways to refine ChatGPT's functionality, such as incorporating more patient-specific data and refining its ability to provide nuanced recommendations based on clinical context. Future ChatGPT interface could also introduce the ability to read medical imaging, such as pre- and post-operative brain MRI which could improve diagnostic ability and treatment recommendations.  Finding ways to remove paywalls surrounding the newest scientific trials may also allow ChatGPT to integrate the newest scientific findings into its decision-making. | N/A |
| Yeung et al. 2023 | April 2023 | AI chatbots not yet ready for clinical use | Pre-print research article; Front Digit Health | To compare the performance of ChatGPT vs Foresight GPT in providing differential diagnoses for clinical vignettes. | Vignettes were provided to ChatGPT and Foresight, and the models were asked to produce the 5 most likely diagnoses. Five clinicians scored the relevancy of each forecasted output, and also recorded whether crucial diagnoses were missing. | Foresight had slightly higher performance compared to ChatGPT for relevancy (93% vs 93% for relevancy in top-1, 83% vs 78% in the top-5). Further, 60% of outputs from ChatGPT had at least one crucial missed diagnosis.  Overall, ChatGPT produces more superficial high-level disease categories instead of specific conditions (e.g. cardiac arrhythmia), while Foresight outputs more specific suggestions as diagnostic codes (e.g. RBBB). LLMs are also prone to "hallucinating", "Falsehood Mimicry" (where ChatGPT sometimes provided outputs that fit a user's assumption rather than asking clarifying questions or providing factual corrections.) Foresight has a more transparent output with saliency maps and level of uncertainty associated with each differential diagnoses. | Transformer-based chatbots are not ready for clinical practice. Future health-related AI chatbots need to be trained via domain-specific data (i.e. real world healthcare data and medical guidelines), fine tuning (e.g. reinforcement learning from human feedback) by expert clinicians and produce transparent outputs. These models should also target more "skilled" end-users (i.e. the healthcare provider and not the patient). | N/A |
| Kim 2023 | Dec 2022 | Search for Medical Information and Treatment Options for Musculoskeletal Disorders through an Artificial Intelligence Chatbot: Focusing on Shoulder Impingement Syndrome | Pre-print research article; still a preprint article Dec 2023 | To assess ChatGPT's ability to provide medical information and treatment options for shoulder impingement syndrome (SIS). | Eight questions were entered into ChatGPT. No quantitative analysis was conducted; ChatGPT responses were provided in the main manuscript. | ChatGPT responded to all requests without any major grammatical or expressional errors. Overall, answers were suitable to the input messages. While most of the content were produced in laymen's terms, some jargons used by medical experts were also included. | NR | N/A |
| Mehnen et al., 2023 | April 2023 | ChatGPT as a medical doctor? A diagnostic accuracy study on common and rare diseases. | Pre-print research article; Pediatric Discovery | To assess ChatGPT 3.5 vs Chat GPT 4’s ability to diagnose common and rare cases | 50 clinical vignettes (40 common and 10 rare cases) were tested on ChatGPT by asking “what are the 10 most likely diagnoses for this patient”. Output was prompted a total of 3 times in independent chats. Answers compared to 3 physicians’ responses. | For common cases, ChatGPT 4 had the most correct diagnoses within its top 10 differentials for each case, followed by ChatGPT 3.5 and then physicians. For rare cases, ChatGPT-4 had the most percentage accurate, followed closely by GPT3-5 and physicians | All analyses and results from GPT should be confirmed with a human reviewer. | N/A |
| Knebel et al. 2023 | April 2023 | Assessment of ChatGPT in the preclinical management of ophthalmological emergencies - an analysis of ten fictional case vignettes | Pre-print research article; still a pre-print in Dec 2023 | To evaluate ChatGPT’s diagnostic and triage accuracy, along with appropriateness of recommended measures for ophthalmological emergencies | 10 case vignettes over a hierarchy of urgency based on acute ophthalmological symptoms were prompts for ChatGPT and given 5 times in independent chats. Answers analyzed by authors. | Accuracy was high and there were appropriate preclinical measures provided; however, there were aso answers that could cause harm to patients. | One limitation of the study is that prompts used were standardized and carefully worded - therefore, it may be best to also test with scenarios that possess difficult, confounding information for the sake of determining whether GPT’s accuracy still stands despite such additional context. | N/A |
| Gravel et al. 2023 | March 2023, officially published Sept 2023 | Learning to fake it: limited responses and fabricated references provided by ChatGPT for medical questions | Pre-print research article; MCP: Digital Health | To evaluate the responses and specifically, the references, provided by ChatGPT | 20 medical questions were given to ChatGPT, and ChatGPT was further prompted to provide references for its answers | There were both major and minor factual errors in ChatGPT’s responses, and many of the fabricated citations appears credible | Suggested investigations into whether the wording of the prompts (i.e. asking ChatGPT to provide published articles and not creating false references). Suggest having experts in a field evaluating responses, alongside multiple raters | N/A |
| Xie & Wang, 2023 | April 2023 | Faithful AI in Healthcare and Medicine | Pre-print research article; still a pre-print article in Dec 2023 | To elucidate why there are factual inconsistencies and hallucinations from LLMs. | A systematic review was conducted with keywords including, “faithful biomedical language modules; mitigation methods and evaluation metrics: factuality/faithfulness/hallucination” | LLMs not trained on medical data, therefore they are not able to fully cover medical knowledge appropriately and effectively. Domain-specific language models like BioBERT, PubmedBERT, BioGPT still possess limited scale as well. | Require investigation into the evaluation of LLMs’ ability to complete tasks beyond question answering and report summarization; for instance, evaluating its ability to create medical text de novo. Should also expand LLMs’ capacities to be multilingual and multimodal. A unified factuality evaluation/standard should be proposed instead of solely manual human evaluation (which takes significant time and resources) | N/A |
| Perlis 2023 | April 2023 | Research Letter: Application of GPT-4 to select next-step antidepressant treatment in major depression | Research letter; still a pre-print article in Dec 2023 | To assess Chat-GPT-plus’s ability to prescribe antidepressants accurately and appropriately in comparison to expert consensus. | Chat GPT-plus was presented with 10 antidepressant vignettes in randomized order and compared to an expert panel’s responses | In 76% of responses, one of the optimal medication choices was always included. However, one of the contraindicated medications or poor choices was included in almost half of the responses. Chat GPT-plus was also able to provide a rationale for its treatment selections. | Require further study into the implications of “caveats” and “warnings” provided by LLMs to users, such as recommendation to be wary of applying any responses it gives clinically without checking with a physician. | 5 |
| Rau et al. 2023 | April 2023; officially published July 2023 | A context-based chatbot surpasses trained radiologists and generic ChatGPT in following the ACR appropriateness guidelines | Pre-print research article; Radiology | To evaluate the potential of using ChatGPT to provide clinical recommendations based on radiological imaging and the American College of Radiology appropriateness criteria. | 50 clinical radiological images were provided to both accGPT (a context-enriched chatbot), ChatGPT-3.5, ChatGPT-4.0, and radiologists | All GPT models performed at minimum, at physicians’ levels. There was significantly reduced time and cost savings when using GPT models to make clinical decisions in comparison to physicians. | Further explore the development of AI chatbots to improve efficiency and time spent in making clinical decisions, along with reducing inappropriate imaging procedures. | N/A |
| Comrie 2023 | April 2023 | ChatGPT Decision Support System: Utility in Creating Public Policy for Concussion/Repetitive Brain Trauma Associated with Neurodegenerative Diseases | Pre-print research article; still a pre-print in Dec 2023 | To determine whether ChatGPT can be used to create policies for concussion and repetitive brain trauma to prevent further neurodegenerative risks associated with such traumas. | Prompts where there is current controversy related to traumatic encephalopathy were inputted, and evaluation was based on human reviewers who would compare ChatGPT’s responses to the terms of reference by legislatures, along with testing what ChatGPT understands are its limitations in self-knowledge, and finally, transcripts of interactions between ChatGPT and government panel were evaluated with human reviewers. | Pros of ChatGPT include that it is able to create tailored responses to prompts that are otherwise controversial and often-avoided topics with policymakers. ChatGPT is also able to confirm its shortcomings when prompted about them.  Cons include that ChatGPT engages in “mirroring”, where the response is similar to the original inputted prompt (aka textual schizophrenia), and ChatGPT did not understand the humour, sarcasm, and wit sometimes included in speeches that it was asked to analyze between policymakers. | Future studies could use a similar method and analysis to test whether ChatGPT can be used to help create public policies across other disciplines and medical issues. | N/A |
| Wagner & Ertl-Wagner, 2023 | April 2023 | Accuracy of Information and References Using ChatGPT-3 for Retrieval of Clinical Radiological Information | Research Article; Can Assoc Radiol J. | Assess accuracy of ChatGPT-3 when answering questions from the daily routine of radiologists. | 88 radiology questions were inputted into ChatGPT. Response accuracy was rated using a 5-point Likert scale. ChatGPT is also asked to generate citations, which was rated using a 5-point Likert scale for relevance. | 59 responses were correct (67%), 15 largely correct (17%), 6 half correct (7%), 4 mostly incorrect (4.5%), and 4 incorrect (4.5%). For citations, 124 references (36.2%) were real out of 343. 47 references (37.9%) were relevant, 2 (1.6%) were mostly relevant, 5 (4%) were half relevant, 7 (5.6%) were mostly irrelevant, 16 (12.9%) were irrelevant. | Explore methods to verify the accuracy and robustness of medical information presented by ChatGPT. | N/A |
| Williams & Shambrook, 2023 | April 2023 | How will artificial intelligence transform cardiovascular computed tomography? A conversation with an AI model | Preprint Research Article; J. Cardiovasc. Comput. Tomogr. | Explore ChatGPT responses to important points of debate in cardiovascular CT. | ChatGPT was given debate questions from the Society of Cardiovascular Computed Tomography 2023 programme and questions about high risk plaque, quantitative plaque analysis, and how AI will transform cardiovascular CT. | Responses contained pros and cons sides of the argument, but the answers were mostly overviews and lacked specific evidence. | N/A | N/A |
| Ueda et al., 2023 | May 2023 | Evaluating GPT-4-based ChatGPT's Clinical Potential on the NEJM Quiz | Preprint Research Article; still a pre-print in Dec 2023 | Assessed ChatGPT’s ability to answer the NEJM quiz. | ChatGPT-4 was given questions from the NEJM quiz, with or without answer choices, and physicians assessed whether GPT answers are similar to the ground truth. | Overall, ChatGPT correctly answered 87% (54/62) of questions without being given answer choices. Accuracy increased to 97% (60/62) after excluding quizzes that required images. The best performing question category was diagnosis. Model accuracy generally improved when given answer choices. | Future studies should focus on expanding the range of clinical scenarios, assessing ChatGPT impact on actual clinical outcomes and provider workload, and exploring ChatGPT performance in different language settings and healthcare environments. Future models should also incorporate image analysis. | N/A |
| Gabriel et al., 2023 | June 2023 | How large language models can augment perioperative medicine: a daring discourse. | Research Article; Reg Anesth Pain Med. | To review the potential of LLMs in perioperative medicine. |  | The authors discuss the potential benefits of large language models in perioperative medicine, including improved clinical decision support, surveillance tools, and documentation quality. They also acknowledge the limitations of this technology, such as the potential for bias and the need for careful validation. | Investigate the use of large language models in different types of pain medicine practices and determine whether their use truly improves documentation performance and/or compliance. | N/A |
| Liao et al., 2023 | June 2023 | Revolutionary Potential of ChatGPT in Constructing Intelligent Clinical Decision Support Systems. | Research Article; Ann Biomed Eng. | To investigate the potential of ChatGPT as a collaborative design tool with human experts in the development of intelligent clinical decision support systems. | Reviewing the literature on the use of ChatGPT in clinical decision support systems. | By collaborating with human expertise, ChatGPT has the potential to revolutionize the development of robust intelligent clinical decision support systems. | NR | N/A |
| Ravipati et al. 2023 | June 2023 | The role of artificial intelligence in dermatology: the promising but limited accuracy of ChatGPT in diagnosing clinical scenarios. | Research Letter; Int J Dermatol. | To examine the accuracy of ChatGPT in diagnosing clinical scenarios in dermatology | ChatGPT was used to predict the primary diagnosis and differentials for 32 clinical scenarios in dermatology. | The study found that while ChatGPT shows promise, its accuracy is limited and it cannot be relied upon as a sole diagnostic tool. | Further research is needed to improve the accuracy of AI in dermatology and to explore how AI technology can be used in conjunction with other diagnostic tools. | 5 |
| Snoswell et al., 2023 | March 2023 | Augmenting intelligence: Augmenting telehealth with large language models. | Research Article; J Telemed Telecare | To explore the potential of large language models (LLMs) in telehealth. | N/A | The authors discuss the rapid uptake of telehealth during the COVID-19 pandemic and the potential of LLMs to improve the efficiency and accuracy of telehealth consultations. They also highlight the need for digital and health literacy among both clinicians and consumers to ensure the accuracy of information provided by LLMs. | The authors suggest further research into the use of LLMs in telehealth, including the development of guidelines for interactions with LLMs in the clinical environment and the improvement of automated tools for checking LLM-generated text for accuracy. They also suggest exploring the potential of LLMs to improve health outcomes for underserved populations. | 5 |
| Gleb et al. 2022 | Jan 2022 | Length of Stay Prediction in Neurosurgery with Russian GPT-3 Language Model Compared to Human Expectations. | Research Article; Stud Health Technol Inform. | Estimate neurosurgery LOS with GPT-3 and compare with physician’s and patient’s LOS predictions. | ruGPT-3 fine-tuned to predict LOS using EHR. GPT-3 LOS predictions on prospective cases compared to physician and patient predictions. | Mean absolute error differences between GPT-3, patients, and physician predictions are NS. Predictions from GPT-3, patients, and physicians weakly and significantly correlate with each other (p<0.01 for all correlations). | Further investigation into the use of ChatGPT to complete modern tasks like predicting length of stay. | N/A |
| Takanobu et al. 2023 | Feb 2023 | Diagnostic Accuracy of Differential-Diagnosis Lists Generated by Generative Pretrained Transformer 3 Chatbot for Clinical Vignettes with Common Chief Complaints: A Pilot Study. | Research Article; Int J Environ Res Public Health | Assess accuracy of differential diagnoses provided by ChatGPT for internal medicine vignettes. | ChatGPT was used to provide differential diagnoses for physician-developed vignettes involving common conditions. Diagnostic accuracy was compared to physician answers. | ChatGPT had an 83.3% accuracy, compared to 98.3% for physician answers. | GPT-3 can be further adjusted via hyperparameter to better suit eHealth diagnoses, and models need to be more transparent and have access to the latest algorithms. Future studies should test more complex cases. | N/A |
| Liu et al. 2023 | Feb 2023 | Assessing the Value of ChatGPT for Clinical Decision Support Optimization. | Preprint Research Article; still a pre-print in Dec 2023 | To test ChatGPT’s ability to answer clinical questions, and to compare its responses against human suggestions. | ChatGPT, four physicians, and one pharmacist were given clinical questions (including queries about CDSS alerts and medication restrictions for certain diagnoses) to answer. Responses were evaluated by clinicians. | ChatGPT responses were rated as mostly similar to humans in terms of understandability, relevance, bias, inversion, and redundancy to CDSS alert logic, but had lower usefulness and acceptability compared to human responses (p<0.001 for both ). | Large language models should be fine-tuned and trained using clinical notes, PubMed articles, and clinical resources such as UpToDate. | N/A |
| Tripathy et al. 2023 | Jan 2023 | Natural Language Processing for Covid-19 Consulting System | Research Article; Procedia Comput. Sci. | To create a COVID chatbot that helps patients to recognize symptoms and receive appropriate treatment. | BERT-GPT and GPT are fine-tuned using the CovidDialog-English dataset to create a virtual platform for COVID-19 consultation. The models are compared to a simple transformer model. | Fine-tuned BART and GPT models outperformed a simple transformer model during human evaluations. BART was the most efficient due to its larger dataset. The AI responses were human-like and clinically relevant. | NR | N/A |
| Levine et al. 2023 | Feb 2023 | The Diagnostic and Triage Accuracy of the GPT-3 Artificial Intelligence Model | Pre-print research Article; still a pre-print in Dec 2023 | To compare GPT-3's diagnostic and triage performance to attending physicians and lay adult internet users. | Internet users and physicians were randomly given vignettes with simple and complex cases and asked to triage & diagnose the cases. GPT-3 was used to generate triage recommendations and differential diagnoses for vignettes after one-shot learning. GPT’s diagnosis was marked correct if it appears within the top 3 in the list. | GPT-3 produced more accurate diagnoses (88%) than lay internet users (54%) but worse than physicians (95%). Its triage accuracy is similar to internet users (71% vs. 74%) but lower than physicians (91%). | GPT-3’s diagnostic and triage abilities should be further assessed. The optimal prompting strategies should be determined.  GPT-3 could also be used to support order entry, documentation, and other language-based tasks. | N/A |
| Harskamp & Clercq 2023 | March 2023 | Performance of ChatGPT as an AI-assisted decision support tool in medicine: a proof-of-concept study for interpreting symptoms and management of common cardiac conditions (AMSTELHEART-2) | Preprint research article; still a pre-print article in Dec 2023 | To evaluate the accuracy of ChatGPT's recommendations on medical questions related to common cardiac symptoms or conditions. | For the clinical case vignettes, two investigators compared output from ChatGPT against actual advice provided by the physicians or expert cardiologists. For the multiple choice questions, the ChatGPT answers were compared against the actual answers. Twenty vignettes obtained via random sampling of cases with possible cardiac symptoms or cardiac work-up/treatments at a community health center. Age, sex, and comorbidities were altered to strip patient identifiers and produce fictionalized cases.  Additionally, 50 multiple-choice questions on various cardiovascular topics were sourced from Medscape and entered into ChatGPT for evaluation. | In patient-physician consultations, ChatGPT's response matched the actual physician's response in 90% of cases, indicating a high level of agreement.  For more complex GP-expert cardiologist consultations, ChatGPT's response matched expert advice in 50% of cases, with 20% being partial matches.  For multiple choice questions, ChatGPT correctly answered 74% of the questions. GPT-3's diagnostic accuracy was higher than that of lay individuals and close to that of physicians. However, its triage accuracy was inferior to that of physicians.  The paper raises concerns about biases in AI models trained on internet text and the need for further research on prompting strategies for models like GPT-3 in clinical settings. | NR | N/A |
| Guo et al. 2023 | Feb 2023; officially published Oct 2023 | neuroGPT-X: Towards an Accountable Expert Opinion Tool for Vestibular Schwannoma | Preprint research article; AANS | To develop and test a context-enriched GPT model for neurosurgery and compare its performance against ChatGPT | Fifteen general questions involving anatomy, surgical management, imaging, clinical contexts, and genetic predispositions were answered by ChatGPT, neuroGPT-X, and four experienced neurosurgeons. Three independent neurosurgeons blindly evaluated the responses. Evaluation criteria included accuracy, coherence, relevance, thoroughness, and overall rating on 0-4 Likert scale. Evaluators were also asked whether they thought the responses were provided by experts or by the GPT models. After blinded evaluations, experts and evaluators were unblinded and asked about satisfaction and clinical value of the GPT models. | Both GPT models answered significantly faster than expert neurosurgeons (P<0.01; 16-49s vs 30-150s). Responses from both GPT models were non-inferior and often superior to expert responses (P<0.0001).  Both GPT models provided responses that are similar, if not higher, quality compared to expert neurosurgeons. Most responses from the GPT models were rated as having "minimal inaccuracies", while neurosurgeon responses were rated to have "some inaccuracies" to "minimal inaccuracies". | Tools like neuroGPT-X can support decision-making by providing standardized processes and assisting practitioners with point-of-care informational aids.  A promising area of development is multi-modal LLMs, which can interpret images, text and other sensory data. This is suitable for fields like neurosurgery, which rely heavily on multiple information modalities.  Data security and confidentiality need to be considered when using LLMs in healthcare. | N/A |
| Noaeen et al. 2023 | Feb 2023 | Unlocking the Power of EHRs: Harnessing Unstructured Data for Machine Learning-based Outcome Predictions | Research article; Annu Int Conf IEEE Eng Med Biol Soc. | To assess ChatGPT's ability to identify the presence of mental health issues in unstructured clinical notes. | A sample of 1058 patient records from an intensive care database were selected and assigned a binary mental health label (0 or 1) by experienced research assistants.  GPT API was provided unstructured notes from the database. | Agreement was 77% between the GPT model and the expert research assistants. Majority of agreements were in the negative category; only 5/111 positive cases were identified as including mental health issues. | Further adjustments to the model needed for higher accuracy.  Intricacy of detecting mental health and the varied nature of mental health concepts and presentation could account for the differences in accuracy between clinical note analyses. | N/A |
| Ayers et al., 2023 | June 2023 | Comparing Physician and Artificial Intelligence Chatbot Responses to Patient Questions Posted to a Public Social Media ForumResearch Article | Research article; JAMA Intern Med. | To evaluate the ability of an AI chatbot assistant (ChatGPT) to provide quality and empathetic responses to patient questions . | The study used a public and nonidentifiable database of questions from a public social media forum (Reddit’s r/AskDocs) to randomly draw 195 exchanges where a verified physician responded to a public question. Chatbot responses were generated by entering the original question into a fresh session on December 22 and 23, 2022. The original question along with anonymized and randomly ordered physician and chatbot responses were evaluated in triplicate by a team of licensed health care professionals . | Evaluators preferred chatbot responses to physician responses in 78.6% of the evaluations. Mean physician responses were significantly shorter than chatbot responses (52 words vs 211 words) . | The paper does not explicitly mention suggested areas of further research. However, further research could explore the use of AI chatbots in other healthcare settings or for other types of patient interactions. | N/A |
